# Genome-Wide Association Study Reveals Candidate Genes for Flowering Time Variation in Common Bean (*Phaseolus vulgaris* L.)

**DOI:** 10.3389/fpls.2019.00962

**Published:** 2019-07-24

**Authors:** Lorenzo Raggi, Leonardo Caproni, Andrea Carboni, Valeria Negri

**Affiliations:** ^1^Dipartimento di Scienze Agrarie, Alimentari e Ambientali (DSA3), Università degli Studi di Perugia, Perugia, Italy; ^2^CREA Research Centre for Cereal and Industrial Crops, Bologna, Italy

**Keywords:** *Phaseolus vulgaris* L., flowering time control, ddRAD-seq, GWAS, candidate gene analysis

## Abstract

The common bean is one of the most important staples in many areas of the world. Extensive phenotypic and genetic characterization of unexplored bean germplasm are still needed to unlock the breeding potential of this crop. Dissecting genetic control of flowering time is of pivotal importance to foster common bean breeding and to develop new varieties able to adapt to changing climatic conditions. Indeed, flowering time strongly affects yield and plant adaptation ability. The aim of this study was to investigate the genetic control of days to flowering using a whole genome association approach on a panel of 192 highly homozygous common bean genotypes purposely developed from landraces using Single Seed Descent. The phenotypic characterization was carried out at two experimental sites throughout two growing seasons, using a randomized partially replicated experimental design. The same plant material was genotyped using double digest Restriction-site Associated DNA sequencing producing, after a strict quality control, a dataset of about 50 k Single Nucleotide Polymorphisms (SNPs). The Genome-Wide Association Study revealed significant and meaningful associations between days to flowering and several SNP markers; seven genes are proposed as the best candidates to explain the detected associations.

## Introduction

Achieving food security is one of the most important challenges to face in the next three decades. FAO’s 2017 prospects’ revision on the world’s population growth reports an expected growth of the population of more than 2 billion people by 2050 (United Nations, 2017). Accordingly, the demand of food will increase, especially in the areas of the world where most of the developing countries are located, mainly in the African continent (Jensen et al., 2012).

In this context, grain legumes are generally regarded as key commodities for improving food security as they are a relatively inexpensive source of amino acids and other important nutrients such as minerals, when compared to livestock and dairy products (Jensen et al., 2012). In addition, due to their ability to fix atmospheric nitrogen, legumes can generally help reducing the use of fertilizers, thus the environmental impact of agriculture (Reay et al., 2012; Andrews and Andrews, 2017). For all these reasons the use of legumes as a key ingredient for a sustainable agricultural production system is at the core of agricultural policy debates in different countries (Zander et al., 2016).

Among grain legumes, common bean (*Phaseolus vulgaris* L., 2*n* = 2*x* = 22) is one of the most important staples in the world, produced over an area of 18 million hectares with a total production of 12 million tons per year (Akibode and Maredia, 2011; Faostat, 2019). Its production mainly occurs in the sub-Saharan Africa and in many Latin American countries (Petry et al., 2015), where it is critical to nutritional security and farmers income generation (Broughton et al., 2003). The cultivated common bean originated in two centers of diversity, giving rise to two genepools: the Mesoamerican, from Central America and the Andean, from the Andes mountains in South America. Many evidences demonstrated that the two genepools are the result of two independent domestication events that led to many morphological and genetic differences (Singh et al., 1991a, b; Kwak and Gepts, 2009).

Upon the introduction of the common bean in Europe from the Americas, hybridization of the two genepools generated further genetic diversity (Gepts et al., 1988; Zeven, 1997; Angioi et al., 2009; Gioia et al., 2013; Maras et al., 2013), for this reason Europe is considered a secondary center of diversification for this species (Angioi et al., 2010). This process led to the constitution of many European common bean landraces that represent a very important resource for plant breeding. In fact, they have been and still are a useful, sometimes unique, source of favorable alleles for abiotic stress, pest and disease resistances (Esquinas-Alcázar, 1993; Angioi et al., 2010).

Landraces are distinct and variable populations that are characterized by useful agronomical traits and adaptation to the specific environments where they were cultivated for a long time. It is important to stress that landraces differ from historical varieties; in fact, they lack “formal” crop improvement and are closely related to knowledge, habits and uses of the people that have been grown them until present times (Raggi et al., 2013). Even if landraces are excellent raw material for breeding new varieties, the within-population genetic diversity of such materials makes their exploitation in plant breeding challenging. This applies to common bean too where intra-landraces genetic diversity can be rather high (Tiranti and Negri, 2007) while intra-individual heterozygosity rather low (Caproni et al., 2018). Indeed, difficulties may arise in the attempt of associating phenotypic traits of interest with the corresponding genetic determinants when using landraces; the identification of such associations is a fundamental prerequisite for allele mining (Visioni et al., 2013). Therefore, the development of a panel of a manageable number of diverse homozygous common bean genotypes is needed to cope with the above-mentioned limitations (Pignone et al., 2015).

The Single Seed Descent (SSD), initially proposed as a modification of the classical bulk breeding scheme to overcome the problem of natural selection (Goulden, 1939), represent a cost-effective approach to achieve that purpose. Given a certain cross, the application of this method to segregating generations allows to maximize the level of retained genetic variation in relation to cost and labor. SSD consists of passing from a generation to the next one by sowing a single seed from each plant (Brim, 1966). In a self-pollinating species like common bean, SSD can be effectively exploited to generate highly homozygous genotypes starting from single individuals of different landraces (Snape and Riggs, 1975).

Although bi-parental mapping has been successful in identifying many significant Quantitative Trait Loci (QTL) mapped to wide intervals in the common bean genome, our knowledge of genes controlling certain traits is still limited (Johnson and Gepts, 2002; Kelly et al., 2003; Blair et al., 2006, 2011; Miklas et al., 2006; Kwak et al., 2008; Perez-Vega et al., 2010). In fact, the resolution of QTL analysis is generally limited by the number of the recombination events; it means that a QTL can span a few centiMorgans (cM), which can indeed be translated into relatively long physical distances, sometimes containing hundreds of candidate genes (Moghaddam et al., 2016). By contrast, Genome Wide Association Mapping (GWAM) considers much more recombination events by using an association panel of individuals, each of those potentially characterized by a unique recombination history (Visscher et al., 2017). In addition, Genome Wide Association Studies (GWAS), based on very high number of markers, allow to test association of the trait of interest with a large part of the genome of the target species. Due to low cost by data point, high robustness, reproducibility and number in the genome, molecular markers based on Single Nucleotide Polymorphism (SNP) detection are those of election for conducting GWAS.

Currently, different approaches can be used to generate large SNP datasets. For example, high-density SNP arrays are already available for several crops (Hao et al., 2017) including common bean. However, such arrays are often designed starting from a limited number of elite genotypes and can produce biased data when used for characterization of non-elite materials. Next Generation Sequencing (NGS) techniques, that equally produce high number of datapoints, are an interesting alternative as they allow cheap not-biased SNP discovery and genotyping. This approaches have already been used and proven efficient in several crops including wheat, barley and pea (Poland et al., 2012; Liu et al., 2014; Annicchiarico et al., 2017). Moreover, the current availability of reference genomes of several crops (that allows to perform *in silico* simulations to optimize the technique and to map the markers) and of collections of genetically diverse pure lines (that allow to reduce sequence coverage due to the absence of heterozygous loci) makes Next Generation Genotyping (NGG) extremely attractive. Among the possible different NGG strategies (Davey et al., 2011; Barilli et al., 2018) double digest Restriction-site Associated DNA sequencing (ddRAD-seq) was the one of choice for this work. ddRAD-seq is a technique based on a digestion of genomic DNA carried out using two restriction enzymes (instead of a single restriction enzyme as in RAD-seq); the resulting DNA fragments are then ligated to sample-specific barcode adapters for subsequent bulk genotyping on an Illumina platform (Peterson et al., 2012).

Schmutz et al. (2014) published the first reference genome for *P. vulgaris*. This achievement opened novel possibilities for common bean NGG making the use of techniques, such as ddRAD-seq, potentially very effective.

Recently, association studies were carried out on the common bean using different plant materials and genotyping approaches. These studies focused on the search of meaningful association of agronomic traits (Moghaddam et al., 2016), nitrogen fixation (Kamfwa et al., 2015a), resistance to diseases (Perseguini et al., 2016), seed weight (Yan et al., 2017), and some technological traits as cooking time in dry beans (Cichy et al., 2015) with possible genetic determinants involved in their control. In some cases, these studies allowed the identification of candidate genes that can be used to develop new genetic stocks for bean breeding programs.

As flowering time is a key trait determining the production of dry matter and seed yield in many crops such as common bean, its manipulation is a relevant plant breeding target to produce novel varieties that are better adapted to changing climatic conditions (Jung and Müller, 2009). For example, early flowering can be exploited to avoid harsh environmental conditions (e.g., drought and heat) and/or escape pathogen attacks that can both negatively affect seed production (as they occur during/after the seed set stage). On the other hand, late flowering can increase seed yield by extending the vegetative phase and increasing the photosynthate accumulation. A flowering time well-synchronized with target environmental conditions would contribute to the achievement of optimal crop performances.

Extensive studies on floral transition revealed a network of regulatory interactions among genes able to promote or inhibit the phenological transition to the reproductive phase (i.e., flowering). In *Arabidopsis* many of the regulatory genes have been identified and functionally characterized (Putterill et al., 2004; Bäurle and Dean, 2006). Moreover, different species, such as medicago (*Medicago truncatula*) (Pierre et al., 2008, 2011; Laurie et al., 2011), pea (*Pisum sativum*) (Lejeune-Hénaut et al., 2008) and narrow-leafed lupin (*Lupinus angustifolius*) (Ksiazkiewicz et al., 2016; Nelson et al., 2017) have been used to investigate the genetic control of flowering in legumes.

In *P. vulgaris* few studies on flowering time variation and control have been carried out to date. QTL mapping studies detected some genomic regions associated with the trait (Koinange et al., 1996; Blair et al., 2006; Perez-Vega et al., 2010). Raggi et al. (2014) found significant associations between some candidate genes and flowering time variation in a common bean collection. Recently, Kamfwa et al. (2015b) and Moghaddam et al. (2016) identified SNPs significantly associated with days to flowering.

In our study GWAS was used to detect key genomic regions involved in flowering time control. To the purpose, a panel of highly homozygous and diverse common bean genotypes was developed using SSD. Genotypes within the panel were subjected to an extensive genotyping, using ddRAD-seq, and phenotypic characterization carried out in different years and locations.

## Materials and Methods

### Plant Material

**FIGURE 1:**
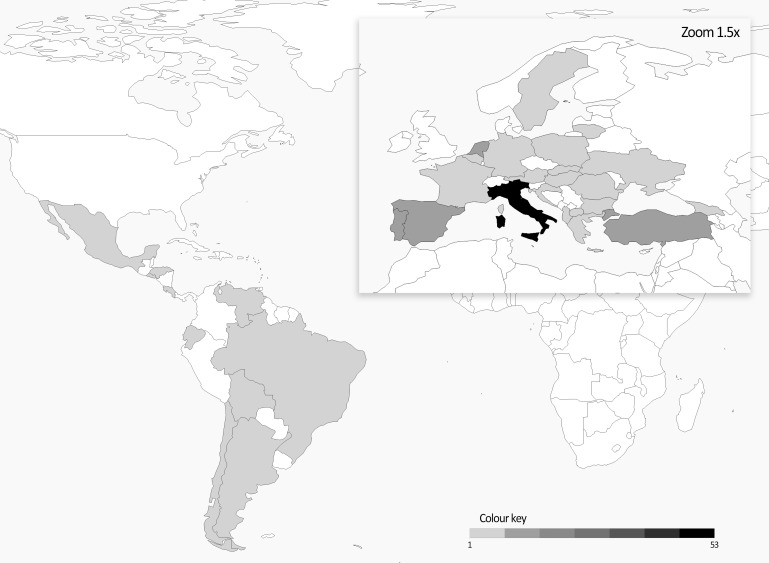
Heatmap of number of accessions per country included in the common bean panel.

The plant material of this work was initially selected with the idea of creating a balanced collection of Andean and Mesoamerican landraces potentially representing an important portion of the European diversity of this species. A similar number of accessions from the two common bean genepools was initially considered; according to the available data of the phaseolin alleles, 97 Andean (57 T + 40 C type) and 84 Mesoamerican (all S type) accessions were included. Europe is the most represented geographical area in the panel (153 accessions) followed by South and Central America (22 and 17, respectively). Italy accounts the highest number of accessions followed by Turkey, Spain, Netherlands, and Portugal. A heatmap representing the origin of the materials is reported in Figure 1.

Starting from the above described collection, 181 common bean highly homozygous genotypes (i.e., pure lines) were obtained applying SSD for at least 5 consecutive generations under isolated conditions. The 181 lines together with 11 cultivars, included as controls, constitute the diversity panel used in this study accounting a total of 192 lines (NCBI BioSample accessions from SAMN12035168 to SAMN12035359). Further details about lines within the panel, including the genebank from which each accession has been originally obtained, are reported in Supplementary Table 1.

### Phenotyping

The phenological characterization of the 192 genotypes was carried out for two consecutive seasons (2016 and 2017) at: (i) DSA3-UNIPG experimental field located in Sant’Andrea d’Agliano, Perugia, Italy (43°3′15.12″N; 12°23′41.64″E, 175 m a.s.l.) (hereafter PG) and (ii) CREA-CI experimental field located in Anzola dell’Emilia, Bologna, Italy (44°34′30.51″N, 11°9′55.64″E, 38 m a.s.l.) (hereafter BO). In 2016 plant material was only evaluated in PG while in 2017 in both PG and BO. In both years sowing has been carried out in May: the 4th (PG_2016), the 11th (PG_2017), and the 12th (BO_2017).

The three experiments were all arranged using partially replicated randomized designs in which five entries were replicated five times and two were replicated six times, producing a total of 222 single plant samples out of 192 entries [total samples = 192 – 7 + (5 × 5) + (2 × 6)]. In PG, the 222 common bean samples were grown in 6 adjacent blocks (fixed size of 1 column × 37 rows) covered by anti-insect net; in BO the same samples were arranged in 3 adjacent blocks (fixed size of 1 column × 74 rows). Plants were grown in a net covered nursery supplied with an automatic drip-irrigation system throughout the entire duration of the trials (May to mid-October). For each sample days to flowering (*dtf*) was recorded as days between sowing and the opening of the first flower (Raggi et al., 2014); a value of 162 days was assigned to the genotypes that did not flower by the end of the experiments (Zhao et al., 2007).

### Phenotypic Data Analyses

The row-column layout of the grown plants and their partial replication allowed for a bi-dimensional spatial analysis of *dtf* (Singh et al., 1997, 2003; Rollins et al., 2013; Raggi et al., 2017). To the purpose, “plot,” “row,” and “column” number was assigned to each sample according to its position. For each entry *dtf* Best Linear Unbiased Predictors (BLUPs) of the genotype effect were calculated in GenStat^®^ (Payne et al., 2011) using the most suitable spatial model determined for the row and column field layout as described by Singh et al. (2003). The procedure consists in gauging the spatial variability by nine applicable models accounting for the existence of different trends, fitting each model (according to the sample position, using the Restricted Maximum Likelihood method, REML), and choosing the best possible one using the Akaike Information Criterion (AIC) (Akaike, 1974). The variance components were used to estimate *dtf* broad-sense heritability $\left(H\,e_{B}^{2}\right)$, along with its standard error, on a plot basis as:

$$H\,e_{B}^{2}=\frac{\sigma_{g}^{2}}{\sigma_{p}^{2}}\times 100$$

where $\sigma_{p}^{2}=\sigma_{e}^{2}+\sigma_{g}^{2}$ (phenotypic variance), $\sigma_{g}^{2}$ = genotypic variance, and $\sigma_{e}^{2}$ = error variance.

Descriptive statistics and Pearson’s correlation coefficients among *dtf* BLUPs of the three trials were calculated using the R package “agricolae” (de Mendiburu, 2017); results were then visualized using “ggplot2” package (Wickham, 2009). BLUPs datasets were then used to perform GWAS.

### DNA Extraction

Genomic DNA was isolated from young leaf tissues, collected from 15 days-old single seedlings, using the TissueLyser II (Qiagen) and the DNeasy 96 plant kit (Qiagen) according to the procedure provided by the manufacturer. DNA concentration and quality were estimated using UV-Vis spectrophotometry (NanoDrop 2000^TM^, Thermo Fisher Scientific). DNA integrity was evaluated after 1% agarose gels (Euro Clone) stained with ethidium bromide electrophoresis. DNA samples were then diluted to 30 ng/μl for following genotyping.

### Genotyping

A double digest Restriction-site Associated DNA sequencing (ddRAD-seq) approach was used for genotyping. The library preparation and the sequencing were carried out by IGAtech (Udine, Italy). Before starting the procedure, a further check of the DNA concertation was produced using a fluorimetric assay to further normalize and uniform the samples. The libraries were produced using a custom protocol (IGAtech), with minor modifications in respect to the one implemented by Peterson and colleagues (Peterson et al., 2012). *In silico* analysis was initially performed to select the best combination of restriction enzymes using the common bean reference genome v1.0 (Schmutz et al., 2014). Since the analysis indicated *Sph*I and *Mbo*I as the best restriction enzymes combination to maximize the number of sequenced loci, they were used for DNA digestion. Digested DNA was purified with AMPureXP beads (Agencourt) and ligated to barcode adapters. Samples were than pooled on multiplexing batches and bead purified. For each pool, target fragment distribution was collected on BluePippin instrument (Sage Instruments Inc., Freedom, CA, United States). Gel eluted fraction was amplified with oligo primers that introduce TruSeq indexes and subsequently bead purified. The resulting libraries were than checked with both Qubit 2.0 Fluorimeter (Invitrogen, Carlsbad, CA, United States) and Bioanalyzer DNA assay (Agilent Technologies, Santa Clara, CA, United States). Libraries were processed with Illumina cBot for cluster generation on the flow cell, following the manufacturer’s instruction and sequenced with V4 chemistry pair end 125 bp mode on HiSeq2500 instrument (Illumina, San Diego, CA, United States).

Demultiplexing of raw Illumina sequences was performed using Stacks v 2.0 (Catchen et al., 2013) and subsequent alignment to the common bean reference genome v 1.0 (Schmutz et al., 2014) using BWA-MEM (Li and Durbin, 2009) with default parameters. Stacks v2.0 was also used to detect all the covered SNP loci from the aligned reads and to filter the detected loci using the population program (included in Stacks v2.0). In this last step, only loci that are represented in at least 75% of the population were retained.

### SNP Quality Control

Several quality control steps were performed on the SNP dataset using PLINK v1.09 (Purcell et al., 2007) and TASSEL v 5.2 software (Bradbury et al., 2007). In particular: (i) SNP loci characterized by values of missingness higher than 10%, (ii) individuals with more than 10% missing loci, and (iii) markers with a Minor Allele Frequency (MAF) lower than 5% were filtered. Loci characterized by heterozygosity ≥2% were also discarded.

### Detection of Population Structure and Cryptic Relatedness

The analyses of structure and cryptic relatedness of genotypes in the panel were carried using a reduced dataset where loci in strong Linkage Disequilibrium (LD) (*r*^2^ ≥ 0.3) were removed. In order to detect the population stratification of the developed panel, a Bayesian clustering approach was used. The number of clusters was initially tested in STRUCTURE v.2.3.4 (Pritchard et al., 2000) assuming an admixture model for different number of clusters (K), ranging from 1 to 11. For each tested cluster 10 iterations were carried out resulting from a 30,000 burn-in period and a Markov Chain Monte Carlo (MCMC) of 30,000 iterations after burn-in. The effective number of clusters was than inferred using the Evanno test (Evanno et al., 2005) implemented in the on-line tool STRUCTURE HARVESTER (Earl and vonHoldt, 2012). According to the result, a new single run was performed at the designed K using 100,000 burn-in period and 200,000 MCMC. The resulting population Q-matrix was used to (i) generate the corresponding Q-plot using the software DiStruct (Rosenberg, 2003) and (ii) to correct the association analyses for the putative population structure. Moreover, a kinship matrix was generated using PLINK v. 1.19 and visualized as heatmap and dendrogram using the R package “ggplot2” (Wickham, 2009).

### Genome-Wide Association Analysis

Marker-trait association analyses were performed using a Mixed Linear Model (MLM) implemented in TASSEL v 5.2 that includes corrections for both population structure (Q) and kinship (K). In fact, the use of such model was necessary as *P. vulgaris* is characterized by a strong genetic structure (Kwak and Gepts, 2009; Raggi et al., 2013). The three BLUP datasets were used as phenotype input matrix in a single association analysis.

The resulting *p-*values were then plotted, as –log_10_(*p*) to produce a Manhattan plot using the R package “CMplots” (Yin, 2016). The correction for multiple-testing was carried out using the Bonferroni adjustment based on the estimated number of independent recombination blocks calculated using PLINK according to Gabriel et al. (2002). For the SNP markers that remained significant after the application of Bonferroni correction, possible candidate genes were identified based on proximity (maximum ± 100 kb) (Patishtan et al., 2018) and by browsing the *P. vulgaris* genome using the online tool Jbrowse on Phytozome v. 12.1 (Goodstein et al., 2012). In order to take advantage of the latest version of the common bean reference genome, sequences containing the significant SNP were positioned against the *P. vulgaris* reference version 2.1. Nucleotide sequences of putative candidate genes were translated into the corresponding proteins and used as queries against the *Arabidopsis thaliana* protein database (Araport11 protein sequences) using the online tool BLASTP (AA query, AA db) available at: https://www.arabidopsis.org/Blast/.

### Linkage Disequilibrium

A raw estimation of LD decay was obtained dividing the size of common bean genome (bp) by the number of independent recombination blocks within the panel, calculated according to Gabriel et al. (2002). In order to ascertain whether significant SNPs, and their relative candidate genes, were located on the same recombination blocks, further LD analyses were carried out. In particular, LD patterns were studied within a window of ±1.5 Mb (centered on the significant marker). Such analysis was only performed for those SNPs located outside the identified candidate genes. Markers within the windows surrounding the associated SNPs were generated using PLINK v. 1.09 by sub-setting the whole SNP dataset obtained after QC and then paired with their corresponding *p*-values. Pairwise LD between markers within the windows (*r*^2^) were calculated using HaploView 4.2 (Barrett et al., 2005); the same software was also used to produce graphical representations of the results.

## Results

### Phenotyping

**FIGURE 2:**
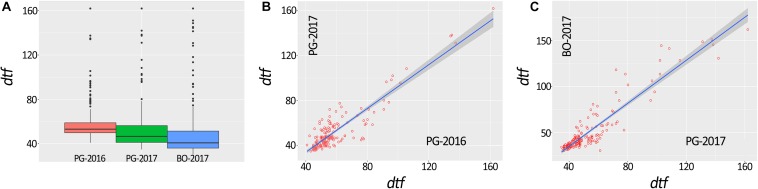
Box plot representation of days to flowering (*dtf*) BLUPs of 192 common bean genotypes **(A)**. Correlation between *dtf* recorded in the same location and different years (i.e., PG-2016 vs. PG-2017) **(B)** and in the same year and different location (i.e., PG-2017 vs. BO-2017) **(C)**.

A total of 648 (97.3%) common bean samples were successfully characterized for *dtf* during the three experiments. In BO-2017, the spatial analysis was more efficient than the completely randomized design with a superior efficiency of the spatial model CrdL (Completely randomized design with linear trends along rows) of 22.4% over the Completely randomized design (Crd); the Crd was the best model for BLUPs calculation from data collected in PG-2016 and 2017. Summary statistics of BLUPs are reported in Table 1. As expected, *dtf* showed high broad sense heritability (He^2^_B_) in all trials (Table 1). Distribution analysis showed consistent data dispersion and the existence of a number of late flowering genotypes (Figure 2A); on the other hand, no differences were observed when data were analyzed separately according to the genepool (Supplementary Figure 1). Simple linear regressions of *dtf* in pairwise comparisons between years (Figure 2B) and experimental sites (Figure 2C) revealed significant and high correlation in both cases with an R^2^ values equal to 0.90 (*P* < 0.001) and 0.93 (*P* < 0.001), respectively. The full BLUPs dataset is available in Supplementary Table 2.

**TABLE 1:** Summary statistics, broad sense heritability, and spatial models used for the estimation of days to flowering BLUPs of 192 common bean genotypes.

	**Mean, days**	**Range, days (Minimum–Maximum)**	**CV (%)**	**He2aB**	**He^2^_B_ SE**	**Model**	**Efficiency (%)**
PG-2016	58.7	40.8–162.0	30.1	0.82	0.051	Crd^b^	100.0
PG-2017	53.1	34.8–162.0	39.3	0.91	0.026	Crd	100.0
BO-2017	50.3	30.2–162.0	52.6	0.94	0.009	CrdL^c^	122.4

*^*a*^Broad sense heritability. ^*b*^Completely randomized design. ^*c*^ Completely randomized design with linear trends along rows.*

### Genotyping

The ddRAD-seq genotyping generated a dataset of 106,072 polymorphic loci, of those 99.3% (105,319) were mapped on the reference genome v.1.0 (Pvulgaris_218_v1.0.fasta) (Schmutz et al., 2014). The full genotyping dataset is available at: https://www.ebi.ac.uk/ena/data/view/PRJEB33063.

**FIGURE 3:**
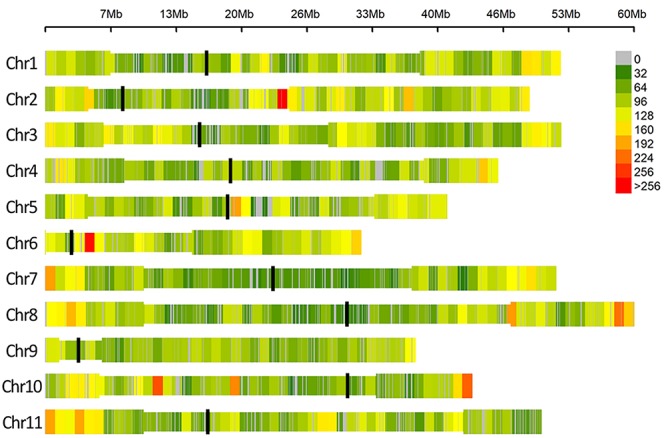
SNP density within 1 Mb window size, different colors represent different density levels. In the Figure “Chr” refers to common bean chromosomes, where centromeric (black line) and pericentromeric regions (thinner bar sections) are reported according to Schmutz et al. (2014).

After quality control, no genotype was excluded and a dataset of 49,518 SNPs markers evenly distributed over the 11 common bean chromosomes was retained for association analyses. A graphical representation of SNPs’ distribution over the eleven bean chromosomes is reported in Figure 3.

### Genetic Structure and Cryptic Relatedness

**FIGURE 4:**
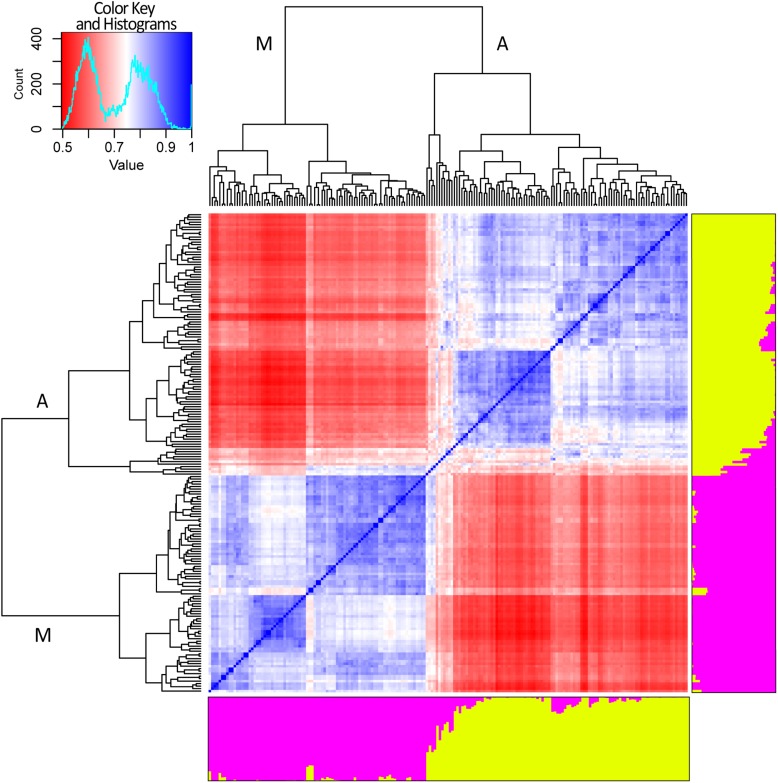
Genetic structure and relatedness among 192 genotypes of common bean. *Genetic structure* (right and bottom side). Each genotype is represented by a column divided into two colored segments (yellow and magenta) whose length indicates the proportions of the genome attributed to each of the two main clusters. *Cryptic relatedness* (center). Heatmap of pairwise similarities between all the genotypes: red, white, and blue for low, medium, and high similarities, respectively. Hierarchical clustering of the panel. The two main groups are indicated with “A” and “M” letters standing for Andean and Mesoamerican, respectively (top and left side).

After removing SNP markers in strong LD (*r*^2^ ≥ 0.3) a dataset of 2,518 SNP was generated and used to perform STRUCTURE and cryptic relatedness analyses (Supplementary Figure 2). Results of the Evanno test clearly indicated *K* = 2 as the most suitable level of population subdivision to explain the genetic structure of the studied panel. STRUCTURE group attributions were strongly consistent with the two common bean genepools (i.e., Mesoamerican and Andean). A graphic representation of the genetic structure of the panel is reported in Figure 4. Considering a threshold of *q* ≥ 0.8 (Bitocchi et al., 2012; Klaedtke et al., 2017), 11 out of 192 genotypes resulted product of admixture between the two genetic groups. All the eleven admixed genotypes derived from European accessions (153) indicating a level of hybridization, between Andean and Mesoamerican genepools, equal to 7.2% (11 out of 153). The admixed entries derived from 9 landrace accessions (Pv_072, Pv_073, Pv_077, Pv_086, Pv_092, Pv_128, Pv_131, Pv_134, and Pv_190) and 2 cultivars (Pv_059 and Pv_064).

Results of cryptic relatedness are also graphically presented in Figure 4. According to genotype origins, inferred using the available information about phaseolins, the blueish square, bottom-left part of the heatmap, includes most of the genotypes of Mesoamerican origin (72). The plot also indicates a further possible sub-structure of the Mesoamerican genotypes into 3 subgroups, the largest of which includes about 50% of all the Mesoamerican samples (Figure 4). On the other hand, the bluish square, top-right part of the heatmap, groups the Andean genotypes (94) with very few exceptions. In this case too, a further subdivision of the group is evident but only one sub-group is clearly distinct (Figure 4). The 11 admixed genotypes are grouped right in the middle of the heatmap; they are characterized by average relatedness values in regard to all other genotypes (light blue, light red, or white color).

### Genome-Wide Association Analysis

**FIGURE 5:**
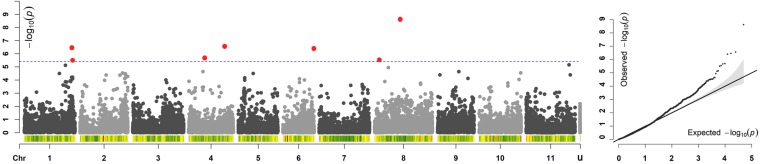
Manhattan and quantile–quantile (Q–Q) plots of *dtf*; In the Manhattan plot, SNPs are ordered by physical position and grouped by chromosome; unmapped SNPs are grouped in “u.” Under each chromosome information on SNP density within 1 Mb window size is also given. The blue dashed line indicates the genome-wide significance threshold. SNPs associated after Bonferroni correction are highlighted in red.

Across all the trials, high and consistent *He*${}_{B}^{2}$ values were observed for *dtf* confirming the suitability of the trait to perform GWAS. The Bonferroni correction for multiple testing, calculated considering the number of independent recombination blocks (2,443), resulted in a threshold equal to 5.4 (–log_10_(p)). GWAS results showed that multiple regions are associated with *dtf* in the common bean genome (Table 2). The lowest *p-*value (i.e., the strongest association) was obtained for SNP 123164_60 on chromosome Pv08. Significant associations were also found for SNPs 66929_307, 17455_7, 95297_22, 59746_63, 59746_36, 116028_71, and 17777_7. In total, 8 significant SNPs for *dtf* were identified in 4 different common bean chromosomes: Pv01, Pv04, Pv06, and Pv08 (Table 2). The Manhattan plot of GWAS results, based on the 49,518 SNP markers, is reported in Figure 5.

**TABLE 2:** List of the significant SNPs identified in the study including physical position, association level, phenotypic variation explained by the SNP and MAF.

**SNP^a^**	**Chromosome**	**SNP position^b^**	***p*-value**	***R*^2^**	**MAF**
123164_60	Pv08	26409992	2.39 × 10^–9^	0.06	*A*(0.50)
66929_307	Pv04	36888939	2.73 × 10^–7^	0.04	*G*(0.39)
17455_7	Pv01	48866257	3.49 × 10^–7^	0.04	*T*(0.48)
95297_22	Pv06	31609022	3.95 × 10^–7^	0.04	*T*(0.42)
59746_63	Pv04	16375177	2.00 × 10^–6^	0.04	*A*(0.38)
59746_36	Pv04	16375150	2.15 × 10^–6^	0.04	*C*(0.38)
116028_71	Pv08	4939572	2.82 × 10^–6^	0.04	*A*(0.46)
17777_7	Pv01	49657488	3.11 × 10^–6^	0.03	*G*(0.10)

*^*a*^SNP names are coded as: “fragment number”_“SNP physical position in the fragment.” ^*b*^SNP physical position on the respective chromosome and according to *P. vulgaris* genome v 1.0.*

### Candidate Gene Identification

The search of possible candidate genes for the most meaningful identified SNP, carried out using Phytozome (v. 12.2) and TAIR, resulted on the identification of 7 possible candidates.

When aligned to the *P. vulgaris* reference genome, the sequenced fragment containing SNP 123164_60 produced multiple hits making the discovery of an associated candidate gene rather complex. However, our analysis detected a relevant gene, *Phvul.008G149900*, located 100 kb upstream of a highly significant hit. Even if no functional annotation was found on the common bean reference genome for this gene, it is noteworthy that its encoded protein is highly similar to *Arabidopsis* At3G12810. This protein, similar to ATP dependent chromatin-remodeling proteins of the ISWI family, is encoded by *Photoperiod-Independent Early flowering 1* (*PIE1*) that is involved in multiple flowering pathways.

Located only 50 kb downstream of the SNP 66929_307, on Pv04, *Phvul.004G112100* is our best candidate to explain the phenotypic variation associated with this marker. The gene encodes for a NAD(P)-binding Rossmann-fold superfamily protein, carrying out oxidoreductase activity in the chloroplast.

**FIGURE 6:**
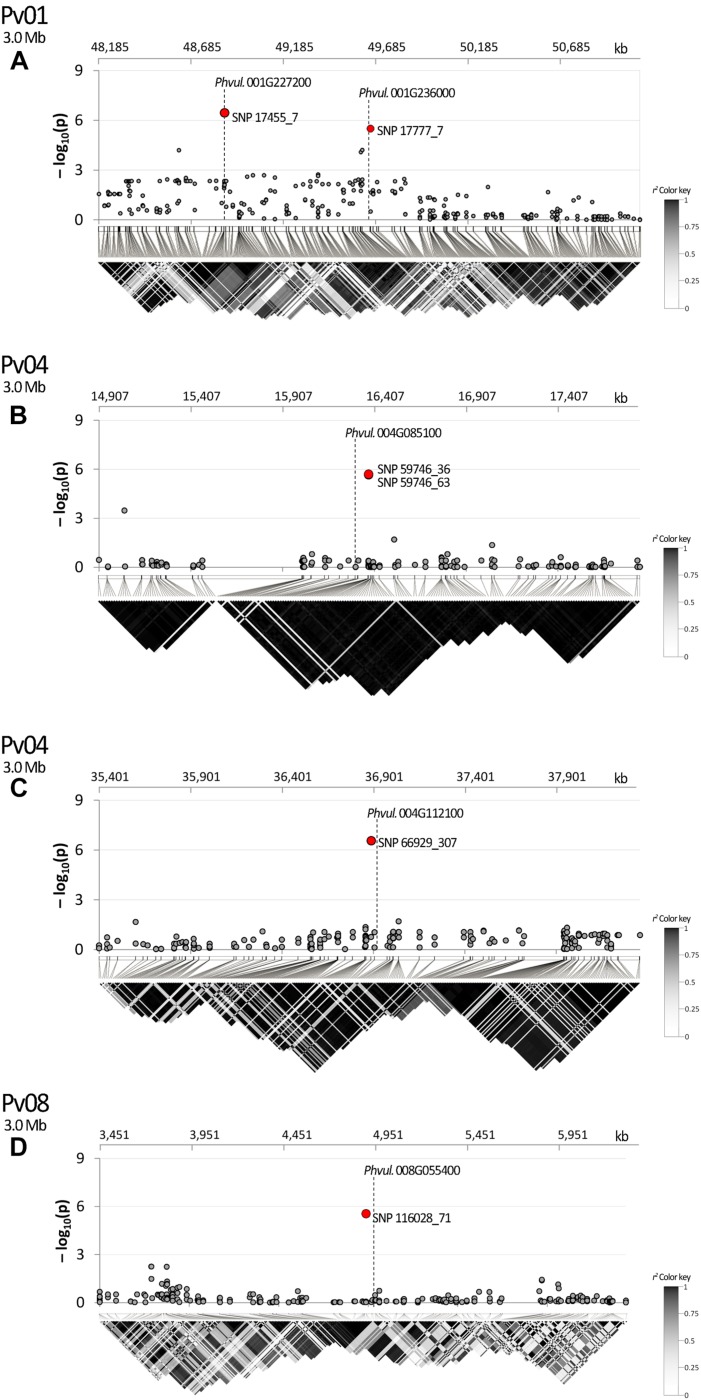
Manhattan plot of *dtf* and LD heatmap over a chromosome region of ±1.5 Mb surrounding SNPs: **(A)** 17777_7; **(B)** 59746_36, 59746_63; **(C)** 666929_307; **(D)** 116028_71. SNPs associated after Bonferroni correction are highlighted in red; candidate genes are placed according to their physical position. In the LD heatmap colors are coded according to the *r*^2^ color key.

The search of the best candidate gene for marker SNP 17455_7 resulted in the identification of *Phvul.001G227200*. In this case, the SNP is located in the first intron of the gene. *Phvul.001G227200* is homologous of the *A. thaliana At1G56260*, also known as *Meristem Disorganization 1* (*MD1*) that is required for the maintenance of stem cells through a reduction in DNA damage (TAIR, 2019b). *Phvul.001G236000* is the best candidate to explain the phenotypic variation associated with the second peak observed in the same region on Pv01 (i.e., SNP 17777_7) as displayed in Figure 6A. The gene, located only 10 kb upstream of the SNP, encodes for a protein phosphatase 2C 3-Receptor, involved in abscisic acid signal transduction.

*Phvul.006G215800* resulted as the best candidate to explain the effect of the SNP 95297_22 detected on Pv06. The gene encodes for Potassium Channel AKT2/3.

The two significant SNPs detected on Pv04 (SNP 59746_36 and 59746_63) co-localize on the same chromosome region being separated by 27 bp only. Located 40 kb upstream of the signal, *Phvul.004G085100* is the best candidate gene explaining the effect of the markers. The homolog gene in *Arabidopsis* encodes for a sucrose transporter protein: *AtSUC2.*

Finally, we identified *Phvul.008G055400* as the most meaningful candidate gene associated to the SNP 116028_71. The gene encodes for a *Leucine-Rich Repeat Receptor-Like Protein*, also known as *Clavata2* (*CLV2*).

### Linkage Disequilibrium

In the studied panel, LD decays in an average distance of circa 240 kb. According to the results of LD analysis, SNP 17777_7 and the candidate *Phvul.001G236000* are in the same recombination block showing the association between the marker and the identified gene (Figure 6A). In the same figure section SNP 17455_7 is also displayed due to its position near to SNP 17777_7. In this case LD analysis was not necessary as the marker is physically located in the first intron of the corresponding candidate (*Phvul.001G227200*); it is noteworthy that several recombination events occurred between the two markers. A clear association was also observed for SNPs 59746_36 and 59746_63 with *Phvul.004G085100* (Figure 6B) and for SNP 66929_307 with *Phvul.004G112100* (Figure 6C). A fairly high average *r*^2^ value was observed between *Phvul.008G055400* recombination block and the SNP 116028_71 (Figure 6D).

## Discussion

The SSD strategy used in this study allowed to produce a panel of highly homozygous common bean genotypes starting from 179 different landraces each of which putatively characterized by relatively high levels of diversity (Tiranti and Negri, 2007; Negri and Tiranti, 2010). Indeed, molecular data demonstrated that the genotypes in our panel are genetically uniform with a very low level of heterozygosity. At the same time, the panel retained a high level of among-genotypes diversity due to the different origin of the initially selected landraces (Figure 4). This approach allowed to build a panel of common bean pure lines that can be indefinitely used for association analyses on a plethora of traits of interest for both basic biology studies as well as for plant breeding. Sample seeds of each developed pure lines are currently conserved, using long-term storage conditions, in the genebank held by DSA3 (FAO code: ITA-363).

Results of the phenotypic characterization for *dtf* showed a rather high level of diversity within the panel (Rodiño et al., 2003; Raggi et al., 2014; Rana et al., 2015). Results of the partially replicated experimental design indicated high levels of *dtf He*${}_{B}^{2}$ that is a crucial parameter to find meaningful and promising associations. Indeed, such design has been already used on barley for association analysis on yield performance (Al-Abdallat et al., 2017). In our study, the use of such experimental design also allowed to test, and to possibly correct, the existence of any bias related to the sample position within the experimental plots such as soil fertility and light exposure. As expected, Crd was the best model for BLUP calculation in two out of three cases since biases were not detected. It is also noteworthy that this particular experimental design allowed to maximize the number of phenotypic datapoints and, at the same time, reducing costs and space needed to characterize such a collection of germplasm.

Among different methods that can be used to generate SNP datasets, the selection of ddRAD-seq approach resulted in a very high number of SNPs evenly distributed over the eleven common bean chromosomes (Figure 3). Regarding the ddRAD-seq used protocol, the *in silico* digestion of the common bean reference genome allowed to select the best enzyme combination maximizing the number of sequenced loci. It is noteworthy that in our study, ddRAD-seq overcame available common bean SNP chips in terms of number of markers successfully genotyped (Cichy et al., 2015; Kamfwa et al., 2015a, b; Moghaddam et al., 2016). In addition, we believe that the technique used for genotyping can help in reducing the ascertainment bias deriving from the use of chip arrays for genotyping of non-elite material.

GWAS is a powerful tool to dissect the genetic control of quantitative traits, potentially providing a higher resolution than QTL mapping. Therefore, in recent years, the interest on this approach arose in both academic and commercial sectors (Davey et al., 2011). In our study, association analysis allowed to detect eight significant SNPs associated with *dtf* on four common bean chromosomes: Pv01, Pv04, Pv06, and Pv08. The analysis of the genomic regions surrounding the detected SNPs allowed the identification of seven meaningful candidate genes that could have an important role in controlling the studied trait. In previous studies, QTL for *dtf* on common bean chromosome Pv01 has been widely reported (Blair et al., 2006; Perez-Vega et al., 2010; Mukeshimana et al., 2014). Moreover, recent research based on GWAS further confirmed the presence of genomic regions involved in the control of this trait on the same chromosome (Kamfwa et al., 2015b; Moghaddam et al., 2016). Similarly, QTL on Pv08 was already reported (Koinange et al., 1996; Perez-Vega et al., 2010) and also confirmed in the above-mentioned studies based on GWAS. According to the mentioned bibliographic records and data produced in our study, the associations on Pv01 and Pv08 are likely to be stable across different environments and genetic background (Kamfwa et al., 2015b). In addition, we observed significant associations on chromosomes Pv04 that were reported by a QTL mapping (Mukeshimana et al., 2014) and GWAS (Moghaddam et al., 2016) study. Finally, Blair et al. (2006) indicated the presence of a QTL for *dtf* on common bean chromosome Pv06 that was also detected in our study.

The first candidate gene identified in this study, *Phvul.008G149900*, encodes for a protein that is highly similar to PIE1. It is noteworthy that mutations of *PIE1* in *A. thaliana* resulted in the suppression of *Flowering Locus C*-mediated delay of flowering and causes early flowering even during non-inductive photoperiods (Noh and Amasino, 2003).

*Phvul.004G112100* resulted the best candidate to explain the phenotypic variation associated with SNP 66929_307. In *A. thaliana* mutants for the homologous gene (*At4G23430*) showed an early-flowering phenotype (TAIR, 2019d) suggesting a role for *Phvul.004G112100* in common bean flowering time control. Mapping homologous Arabidopsis sequences for photoperiod sensitivity in common bean, Kwak et al. (2008) located one of the homolog of *Terminal Flower1* (*PvTFLx*) on chromosome Pv04. In particular, *PvTFLx* is located 2 Mb downstream our best candidate suggesting that this region harbors different genes involved in flowering time control.

*Phvul.001G227200*, homolog of *MD1* in Arabidopsis, was the resulting candidate to explain the significance of the SNP 17455_7. Interestingly, in *A. thaliana* mutants of this gene showed several development defects such as abnormal phyllotaxy and plastochron, stem fasciation and reduced root growth (Hashimura and Ueguchi, 2011). In the same study the authors reported that in mutants “leaves and floral buds did not develop in a spatially and temporally regulated manner” opening for a possible role of the gene in flowering control. It is also noteworthy that, in maize, shoot apical meristem development has been associated with flowering time (Leiboff et al., 2015). Further analyses, within the same chromosomic region of the previous candidate, revealed that *Phvul.001G236000* is the best candidate to explain the phenotypic variation of the marker 17777_7. In *A. thaliana* mutants of the homologous *At4G26080* showed a late flowering phenotype. It is noteworthy that this narrow chromosomic region (circa 1.3 Mb) contains the two candidate genes detected in this study on Pv01 together with *Phvul.001G221100* that encodes for Phytocrome A (Kamfwa et al., 2015b). Finally, this chromosomic region overlaps with a QTL for days to flowering identified by Blair et al. (2006) using a by-parental mapping approach: one of the two flanking markers of the QTL falls in the same above-mentioned chromosomic region. All these experimental evidences suggest the presence of a gene cluster involved in flowering time control in chromosome Pv01.

*Phvul.006G215800*, the best proposed candidate for the marker 9529_7 on chromosome Pv06, encodes for Potassium Channel AKT2/3, a photosynthate and light-dependent inward rectifying potassium channel with unique gating proprieties that are regulated by phosphorylation (TAIR, 2019e). It has been demonstrated that loss of function of *AKT2/3* affects sugar loading into the phloem of *A. thaliana* and mutants show delayed flower induction and rosette development (Deeken et al., 2002). Such phenotype strongly corroborates our hypothesis of the involvement of *Phvul.006G215800* in flowering.

*Phvul.004G085100* on the chromosome Pv04 encodes for a sucrose transport protein. Interestingly, in *A. thaliana*, a mutation of the homolog (*At1G22710*) causes dwarfism, delayed development and it has been reported that such plants can occasionally flower, but never produce viable seeds (TAIR, 2019a). *At1G22710*, also known as *AtSUC2*, was one of the first genes associated with sucrose transporters (Sauer and Stolz, 1994); this gene is required for phloem loading of sucrose and its activity has been described in detail by Chandran et al. (2003). It is well known that sucrose is a relevant element within the flowering induction process (Corbesier et al., 1998); in plants, sucrose is the main form of fixed carbon that is transported in phloem and also serves as specific signaling molecule (Teng et al., 2005; Solfanelli et al., 2006). An increase in carbohydrate export from leaves has been generally associated with floral induction in *Arabidopsis* (Corbesier et al., 1998). Consistently, in *Nicotiana tabacum* L., a decreased phloem loading of sucrose, induced by antisense repression of the *NtSUT1* causes delayed flowering (Burkle et al., 1998). Moreover, in *A. thaliana* sucrose availability on the aerial part of the plant promotes flowering even in dark conditions (Roldán et al., 1999). All these evidences strongly suggest a role of *Phvul.004G085100* in controlling flowering time in *P. vulgaris*. In addition, it is also relevant to mention that Mukeshimana et al. (2014) found two QTLs for days to flowering on the mid-terminal part of Pv04 that might roughly correspond to the chromosome region in which *Phvul.004G085100* is located. However, since in above-mentioned study SNP marker positions are expressed as cM, it is difficult to ascertain whether our candidate falls within these regions.

In conclusion, *Phvul.008G055400*, the candidate gene identified in relation the marker 116028_71 on the chromosome Pv08, encodes for CLV2. In *A. thaliana* a mutation of the homolog of this gene (*At1G65380*) causes altered flower development, late flowering or interrupted flowering caused by a temporary termination of the main inflorescence flower meristem (TAIR, 2019c). In a recent paper, Basu et al. (2019) identified four *Clavata* genes, including *Clavata2*, that are highly associated with days to flowering in *Cicer arietinum* L.

As from the discussed evidences and related bibliographic records, homolog of most of the genes that we propose as candidates for explaining observed flowering time variation in the studied common bean panel are involved in different pathways regulating flowering in *Arabidopsis*, tobacco, maize, and chickpea. Indeed, results of this research are an important step forward in understanding flowering time control in one of the most important pulses world-wide. Although this diversity panel is representative of a large portion of the European common bean diversity, performing similar analyses on a wider and/or more diverse panel would help in confirming the detected associations. In addition, the application of gene knockout to the proposed candidates would further confirm their involvement in the genetic control of flowering time and allow to measure their contribution to its expression under different experimental conditions (e.g., short vs. long day treatments). The exploitation of the genes identified in this research will hopefully allow the development of new common bean varieties able to better adapt to changing climatic conditions.

## Data Availability

NCBI BioSample accessions of the 192 common bean lines from: SAMN12035168 to SAMN12035359. The full genotyping dataset of the same samples is available at: https://www.ebi.ac.uk/ena/data/view/PRJEB33063.

## Author Contributions

VN and LR conceived and designed the experiments. LC and AC performed plant phenotyping. LR, LC, and AC performed phenotypic data analyses. LR and LC performed GWAS and candidate gene data analyses. AC and VN contributed to phenotyping costs. VN funded reagents, materials, and analysis tools. All authors wrote the manuscript.

## Conflict of Interest Statement

The authors declare that the research was conducted in the absence of any commercial or financial relationships that could be construed as a potential conflict of interest.

## References

[B1] Akaike H. (1974). A new look at the statistical model identification. *IEEE Trans. Automat. Contr.* 19 716–723. 10.1109/TAC.1974.1100705

[B2] Akibode S. Maredia M. (2011). Global and regional trends in production, trade and consumption of food legume crops. *Dep. Agric. Food Resour. Econ* 87 1–83.

[B3] Al-Abdallat A. M. Karadsheh A. Hadadd N. I. Akash M. W. Ceccarelli S. Baum M. (2017). Assessment of genetic diversity and yield performance in jordanian barley (*Hordeum vulgare* L.) landraces grown under Rainfed conditions. *BMC Plant Biol.* 17:191. 10.1186/s12870-017-1140-1 29096621 PMC5668982

[B4] Andrews M. Andrews M. E. (2017). Specificity in legume-rhizobia symbioses. *Int. J. Mol. Sci* 18:E705. 10.3390/ijms18040705 28346361 PMC5412291

[B5] Angioi S. A. Desiderio F. Rau D. Bitocchi E. Attene G. Papa R. (2009). Development and use of chloroplast microsatellites in *Phaseolus* spp. and other legumes. *Plant Biol.* 11 598–612. 10.1111/j.1438-8677.2008.00143.x 19538398

[B6] Angioi S. A. Rau D. Attene G. Nanni L. Bellucci E. Logozzo G. (2010). Beans in Europe: origin and structure of the European landraces of *Phaseolus vulgaris* L. *Theor. Appl. Genet.* 121 829–843. 10.1007/s00122-010-1353-2 20490446

[B7] Annicchiarico P. Nazzicari N. Wei Y. Pecetti L. Brummer E. C. (2017). Genotyping-by-sequencing and its exploitation for Forage and cool-season grain legume breeding. *Front. Plant Sci.* 8:679. 10.3389/fpls.2017.00679 28536584 PMC5423274

[B8] Barilli E. Cobos M. J. Carrillo E. Kilian A. Carling J. Rubiales D. (2018). A high-density integrated DArTseq SNP-based genetic Map of *Pisum fulvum* and identification of QTLs controlling rust resistance. *Front. Plant Sci.* 9:167. 10.3389/fpls.2018.00167 29497430 PMC5818415

[B9] Barrett J. C. Fry B. Maller J. Daly M. J. (2005). Haploview: analysis and visualization of LD and haplotype maps. *Bioinformatics* 21 263–265. 10.1093/bioinformatics/bth457 15297300

[B10] Basu U. Narnoliya L. Srivastava R. Sharma A. Bajaj D. Daware A. (2019). CLAVATA signaling pathway genes modulating flowering time and flower number in chickpea. *Theor. Appl. Genet.* 132 2017–2038. 10.1007/s00122-019-03335-y 30929032

[B11] Bäurle I. Dean C. (2006). The timing of developmental transitions in plants. *Cell* 125 655–664. 10.1016/j.cell.2006.05.005 16713560

[B12] Bitocchi E. Nanni L. Bellucci E. Rossi M. Giardini A. Zeuli P. S. (2012). Mesoamerican origin of the common bean (*Phaseolus vulgaris* L.) is revealed by sequence data. *Proc. Natl. Acad. Sci. U.S.A* 109 E788–E796. 10.1073/pnas.1108973109 22393017 PMC3325731

[B13] Blair M. W. Astudillo C. Rengifo J. Beebe S. E. Graham R. (2011). QTL analyses for seed iron and zinc concentrations in an intra-genepool population of andean common beans (*Phaseolus vulgaris* L.). *Theor. Appl. Genet.* 122 511–521. 10.1007/s00122-010-1465-8 21113704

[B14] Blair M. W. Iriarte G. Beebe S. (2006). QTL analysis of yield traits in an advanced backcross population derived from a cultivated andean × wild common bean (*Phaseolus vulgaris* L.) cross. *Theor. Appl. Genet.* 112 1149–1163. 10.1007/s00122-006-0217-2 16432734

[B15] Bradbury P. J. Zhang Z. Kroon D. E. Casstevens T. M. Ramdoss Y. Buckler E. S. (2007). TASSEL: software for association mapping of complex traits in diverse samples. *Bioinformatics* 23 2633–2635. 10.1093/bioinformatics/btm308 17586829

[B16] Brim C. A. (1966). A modified pedigree method of selection in soybeans. *Crop Sci.* 6:220. 10.2135/cropsci1966.0011183X000600020041x 24227141

[B17] Broughton W. Hernandez G. Blair M. (2003). Beans (*Phaseolus* spp.) – model food legumes. *Plant Soil* 252 55–128. 10.1023/a:1024146710611

[B18] Burkle L. Hibberd J. M. Quick W. P. Kuhn C. Hirner B. Frommer W. B. (1998). The H+-sucrose cotransporter NtSUT1 is essential for sugar export from tobacco leaves. *Plant Physiol.* 118 59–68. 10.1104/pp.118.1.59 9733526 PMC34874

[B19] Caproni L. Raggi L. Tissi C. Howlett S. Torricelli R. Negri V. (2018). Multi-Environment evaluation and genetic characterisation of common bean breeding lines for organic farming systems. *Sustainability* 10:777 10.3390/su10030777

[B20] Catchen J. Hohenlohe P. A. Bassham S. Amores A. Cresko W. A. (2013). Stacks: an analysis tool set for population genomics. *Mol. Ecol.* 22 3124–3140. 10.1111/mec.12354 23701397 PMC3936987

[B21] Chandran D. Reinders A. Ward J. M. (2003). Substrate specificity of the *Arabidopsis thaliana* sucrose transporter AtSUC2. *J. Biol. Chem.* 278 44320–44325. 10.1074/jbc.M308490200 12954621

[B22] Cichy K. A. Wiesinger J. A. Mendoza F. A. (2015). Genetic diversity and genome-wide association analysis of cooking time in dry bean (*Phaseolus vulgaris* L.). *Theor. Appl. Genet.* 128 1555–1567. 10.1007/s00122-015-2531-z 26003191

[B23] Corbesier L. Lejeune P. Bernier G. (1998). The role of carbohydrates in the induction of flowering in *Arabidopsis thaliana*: comparison between the wild type and a starchless mutant. *Planta* 206 131–137. 10.1007/s004250050383 9715535

[B24] Davey J. W. Hohenlohe P. A. Etter P. D. Boone J. Q. Catchen J. M. Blaxter M. L. (2011). Genome-wide genetic marker discovery and genotyping using next-generation sequencing. *Nat. Rev. Genet.* 12 499–510. 10.1038/nrg3012 21681211

[B25] de Mendiburu F. (2017). *R**package “Agricolae.”.* 1–153. Available at: http://tarwi.lamolina.edu.pe/f̃mendiburu/ (accessed February 5, 2019).

[B26] Deeken R. Geiger D. Fromm J. Koroleva O. Ache P. Langenfeld-Heyser R. (2002). Loss of the AKT2/3 potassium channel affects sugar loading into the phloem of *Arabidopsis*. *Planta* 216 334–344. 10.1007/s00425-002-0895-1 12447548

[B27] Earl D. A. vonHoldt B. M. (2012). STRUCTURE HARVESTER: a website and program for visualizing STRUCTURE output and implementing the evanno method. *Conserv. Genet. Resour* 4 359–361. 10.1007/s12686-011-9548-7

[B28] Esquinas-Alcázar J. T. (1993). “Plant genetic resources,” in *Plant Breeding: Principales and Prospects*, eds Hayward M. D. Bosemark N. O. Romagosa I. (London, UK: Chapman & Hall), 33–51.

[B29] Evanno G. Regnaut S. Goudet J. (2005). Detecting the number of clusters of individuals using the software STRUCTURE: a simulation study. *Mol. Ecol.* 14 2611–2620. 10.1111/j.1365-294X.2005.02553.x 15969739

[B30] Faostat (2019). *Food and Agriculture Organization of the United Nations.* Rome: FAOSTAT database.

[B31] Gabriel S. B. Schaffner S. F. Nguyen H. Moore J. M. Roy J. Blumenstiel B. (2002). The structure of haplotype blocks in the human genome. *Science.* 296 2225–2229. 10.1126/science.1069424 12029063

[B32] Gepts P. Kmiecik K. Pereira P. Bliss F. (1988). Dissemination pathways of common bean (*Phaseolus vulgaris* L). *Econ. Bot.* 42:86 10.1007/bf02859038

[B33] Gioia T. Logozzo G. Kami J. Spagnoletti Zeuli P. Gepts P. (2013). Identification and characterization of a homologue to the *Arabidopsis* INDEHISCENT gene in common bean. *J. Hered.* 104 273–286. 10.1093/jhered/ess102 23235700

[B34] Goodstein D. M. Shu S. Howson R. Neupane R. Hayes R. D. Fazo J. (2012). Phytozome: a comparative platform for green plant genomics. *Nucleic Acids Res.* 40 D1178–D1186. 10.1093/nar/gkr944 22110026 PMC3245001

[B35] Goulden C. H. (1939). “Problems in plant selection,” in *Proceeding of the Seventh International Genetical Congress*, (Edinburgh), 132–133.

[B36] Hao Y. Xu Y. Khan A. He Z. Rasheed A. Varshney R. K. (2017). Crop breeding chips and genotyping platforms: progress, challenges, and perspectives. *Mol. Plant* 10 1047–1064. 10.1016/j.molp.2017.06.008 28669791

[B37] Hashimura Y. Ueguchi C. (2011). The *Arabidopsis* MERISTEM DISORGANIZATION 1 gene is required for the maintenance of stem cells through the reduction of DNA damage. *Plant J.* 68 657–669. 10.1111/j.1365-313X.2011.04718.x 21781195

[B38] Jensen E. S. Peoples M. B. Boddey R. M. Gresshoff P. M. Henrik H. N. Alves B. J. R. (2012). Legumes for mitigation of climate change and the provision of feedstock for biofuels and biorefineries. *Agron. Sustain. Dev.* 32 329–354. 10.1007/s13593-011-0056-7

[B39] Johnson W. C. Gepts P. (2002). The role of epistasis in controlling seed yield and other agronomic traits in an andean x mesoamerican cross of common bean (*Phaseolus vulgaris* L.). *Euphytica* 125 69–79. 10.1023/A:1015775822132

[B40] Jung C. Müller A. E. (2009). Flowering time control and applications in plant breeding. *Trends Plant Sci.* 14 563–573. 10.1016/j.tplants.2009.07.005 19716745

[B41] Kamfwa K. Cichy K. A. Kelly J. D. (2015a). Genome-wide association analysis of symbiotic nitrogen fixation in common bean. *Theor. Appl. Genet.* 128 1999–2017. 10.1007/s00122-015-2562-5 26133733

[B42] Kamfwa K. Cichy K. A. Kelly J. D. (2015b). Genome-wide association study of agronomic traits in common bean. *Plant Genome* 8 1–12. 10.3835/plantgenome2014.09.0059 33228312

[B43] Kelly J. D. Gepts P. Miklas P. N. Coyne D. P. (2003). Tagging and mapping of genes and QTL and molecular marker-assisted selection for traits of economic importance in bean and cowpea. *F. Crop. Res.* 82 135–154. 10.1016/S0378-4290(03)00034-0

[B44] Klaedtke S. Caproni L. Klauck J. de la Grandville P. Dutartre M. Stassart P. (2017). Short-term local adaptation of historical common bean (*Phaseolus vulgaris* L.) varieties and implications for in situ management of Bean Diversity. *Int. J. Mol. Sci.* 18:E493. 10.3390/ijms18030493 28264476 PMC5372509

[B45] Koinange E. M. K. Singh S. P. Gepts P. (1996). Genetic control of the domestication syndrome in common-bean. *Crop Sci.* 36 1037–1045. 10.2135/cropsci1996.0011183X003600040037x

[B46] Ksiazkiewicz M. Rychel S. Nelson M. N. Wyrwa K. Naganowska B. Wolko B. (2016). Expansion of the phosphatidylethanolamine binding protein family in legumes: a case study of *Lupinus angustifolius* L. FLOWERING LOCUS T homologs, LanFTc1 and LanFTc2. *BMC Genomics* 17:820. 10.1186/s12864-016-3150-z 27769166 PMC5073747

[B47] Kwak M. Gepts P. (2009). Structure of genetic diversity in the two major gene pools of common bean (*Phaseolus vulgaris* L., Fabaceae). *Theor. Appl. Genet.* 118 979–992. 10.1007/s00122-008-0955-4 19130029

[B48] Kwak M. Velasco D. Gepts P. (2008). Mapping homologous sequences for determinacy and photoperiod sensitivity in common bean (*Phaseolus vulgaris*). *J. Hered.* 99 283–291. 10.1093/jhered/esn005 18316323

[B49] Laurie R. E. Diwadkar P. Jaudal M. Zhang L. Hecht V. Wen J. (2011). The medicago FLOWERING LOCUS T homolog, MtFTa1, is a key regulator of flowering time. *Plant Physiol.* 156 2207–2224. 10.1104/pp.111.180182 21685176 PMC3149922

[B50] Leiboff S. Li X. Hu H. C. Todt N. Yang J. Li X. (2015). Genetic control of morphometric diversity in the maize shoot apical meristem. *Nat. Commun.* 6 1–10. 10.1038/ncomms9974 26584889 PMC4673881

[B51] Lejeune-Hénaut I. Hanocq E. Béthencourt L. Fontaine V. Delbreil B. Morin J. (2008). The flowering locus Hr colocalizes with a major QTL affecting winter frost tolerance in *Pisum sativum* L. *Theor. Appl. Genet.* 116 1105–1116. 10.1007/s00122-008-0739-x 18347775

[B52] Li H. Durbin R. (2009). Fast and accurate short read alignment with Burrows-wheeler transform. *Bioinformatics* 25 1754–1760. 10.1093/bioinformatics/btp324 19451168 PMC2705234

[B53] Liu H. Bayer M. Druka A. Russell J. R. Hackett C. A. Poland J. (2014). An evaluation of genotyping by sequencing (GBS) to map the Breviaristatum-e (ari-e) locus in cultivated barley. *BMC Genomics* 15:104. 10.1186/1471-2164-15-4 24498911 PMC3922333

[B54] Maras M. Šuštar-Vozliè J. Kainz W. Megliè V. (2013). Genetic diversity and dissemination pathways of common bean in Central Europe. *J. Am. Soc. Hortic. Sci.* 138 297–305. 10.21273/jashs.138.4.297

[B55] Miklas P. N. Kelly J. D. Beebe S. E. Blair M. W. (2006). Common bean breeding for resistance against biotic and abiotic stresses: from classical to MAS breeding. *Euphytica* 147 105–131. 10.1007/s10681-006-4600-5

[B56] Moghaddam S. M. Mamidi S. Osorno J. M. Lee R. Brick M. Kelly J. (2016). Genome-wide association study identifies candidate loci underlying agronomic traits in a Middle American diversity panel of common bean. *Plant Genome* 9 1–21. 10.3835/plantgenome2016.02.0012 27902795

[B57] Mukeshimana G. Butare L. Cregan P. B. Blair M. W. Kelly J. D. (2014). Quantitative trait loci associated with drought tolerance in common bean. *Crop Sci.* 54 923–938. 10.2135/cropsci2013.06.0427

[B58] Negri V. Tiranti B. (2010). Effectiveness of in situ and ex situ conservation of crop diversity. What a Phaseolus vulgaris L. landrace case study can tell us. *Genetica* 138 985–998. 10.1007/s10709-010-9485-5 20835753

[B59] Nelson M. N. Książkiewicz M. Rychel S. Besharat N. Taylor C. M. Wyrwa K. (2017). The loss of vernalization requirement in narrow-leafed lupin is associated with a deletion in the promoter and de-repressed expression of a Flowering Locus T (FT) homologue. *New Phytol.* 213 220–232. 10.1111/nph.14094 27418400

[B60] Noh Y.-S. Amasino R. M. (2003). PIE1, an ISWI family gene, is required for FLC activation and floral repression in *Arabidopsis*. *Plant Cell* 15 1671–1682. 10.1105/tpc.012161 12837955 PMC165409

[B61] Patishtan J. Hartley T. N. Fonseca de Carvalho R. Maathuis F. J. M. (2018). Genome-wide association studies to identify rice salt-tolerance markers. *Plant Cell Environ.* 41 970–982. 10.1111/pce.12975 28436093

[B62] Payne R. W. Harding S. A. Murray D. A. Soutar D. M. Baird D. B. Glaser A. I. (2011). *The Guide to GenStat Release 14, Part 2: Statistics.* Hemel Hempstead: VSN International.

[B63] Perez-Vega E. Paneda A. Rodriguez-Suarez C. Campa A. Giraldez R. Ferreira J. J. (2010). Mapping of QTLs for morpho-agronomic and seed quality traits in a RIL population of common bean (*Phaseolus vulgaris* L.). *Theor. Appl. Genet.* 120 1367–1380. 10.1007/s00122-010-1261-5 20084493

[B64] Perseguini J. M. K. C. Oblessuc P. R. Rosa J. R. B. F. Gomes K. A. Chiorato A. F. Carbonell S. A. M. (2016). Genome-wide association studies of anthracnose and angular leaf spot resistance in common bean (*Phaseolus vulgaris* L.). *PLoS One* 11:e0150506. 10.1371/journal.pone.0150506 26930078 PMC4773255

[B65] Peterson B. K. Weber J. N. Kay E. H. Fisher H. S. Hoekstra H. E. (2012). Double digest RADseq: an inexpensive method for de novo SNP discovery and genotyping in model and non-model species. *PLoS One* 7:e37135. 10.1371/journal.pone.0037135 22675423 PMC3365034

[B66] Petry N. Boy E. Wirth J. P. Hurrell R. F. (2015). Review: the potential of the common bean (*Phaseolus vulgaris*) as a vehicle for iron biofortification. *Nutrients* 7 1144–1173. 10.3390/nu7021144 25679229 PMC4344581

[B67] Pierre J. B. Bogard M. Herrmann D. Huyghe C. Julier B. (2011). A CONSTANS-like gene candidate that could explain most of the genetic variation for flowering date in Medicago truncatula. *Mol. Breed.* 28 25–35. 10.1007/s11032-010-9457-6

[B68] Pierre J. B. Huguet T. Barre P. Huyghe C. Julier B. (2008). Detection of QTLs for flowering date in three mapping populations of the model legume species *Medicago truncatula*. *Theor. Appl. Genet.* 117 609–620. 10.1007/s00122-008-0805-4 18553068

[B69] Pignone D. De Paola D. Rapanà N. Janni M. (2015). Single seed descent: a tool to exploit durum wheat (*Triticum durum* Desf.) genetic resources. *Genet. Resour. Crop Evol.* 62 1029–1035. 10.1007/s10722-014-0206-2

[B70] Poland J. A. Brown P. J. Sorrells M. E. Jannink J.-L. (2012). Development of high-density genetic maps for barley and wheat using a novel two-enzyme genotyping-by-sequencing approach. *PLoS One* 7:e32253. 10.1371/journal.pone.0032253 22389690 PMC3289635

[B71] Pritchard J. K. Stephens M. Donnelly P. (2000). Inference of population structure using multilocus genotype data. *Genetics* 155 945–959. 10.1111/j.1471-8286.2007.01758.x 10835412 PMC1461096

[B72] Purcell S. Neale B. Todd-Brown K. Thomas L. Ferreira M. A. R. Bender D. (2007). PLINK: a tool set for whole-genome association and population-based linkage analyses. *Am. J. Hum. Genet.* 81 559–575. 10.1086/519795 17701901 PMC1950838

[B73] Putterill J. Laurie R. Macknight R. (2004). It’s time to flower: the genetic control of flowering time. *BioEssays* 26 363–373. 10.1002/bies.20021 15057934

[B74] Raggi L. Ciancaleoni S. Torricelli R. Terzi V. Ceccarelli S. Negri V. (2017). Evolutionary breeding for sustainable agriculture: selection and multi-environmental evaluation of barley populations and lines. *F. Crop. Res.* 204 76–88. 10.1016/j.fcr.2017.01.011

[B75] Raggi L. Tiranti B. Negri V. (2013). Italian common bean landraces: diversity and population structure. *Genet. Resour. Crop Evol.* 60 1515–1530. 10.1007/s10722-012-9939-y

[B76] Raggi L. Tissi C. Mazzucato A. Negri V. (2014). Molecular polymorphism related to flowering trait variation in a *Phaseolus vulgaris* L. collection. *Plant Sci.* 21 180–189. 10.1016/j.plantsci.2013.11.001 24388529

[B77] Rana J. C. Sharma T. R. Tyagi R. K. Chahota R. K. Gautam N. K. Singh M. (2015). Characterisation of 4274 accessions of common bean (*Phaseolus vulgaris* L.) germplasm conserved in the Indian gene bank for phenological, morphological and agricultural traits. *Euphytica* 205 441–457. 10.1007/s10681-015-1406-3

[B78] Reay D. S. Davidson E. A. Smith K. A. Smith P. Melillo J. M. Dentener F. (2012). Global agriculture and nitrous oxide emissions. *Nat. Clim. Chang.* 2 410–416. 10.1038/nclimate1458

[B79] Rodiño A. P. Santalla M. De Ron A. M. Singh S. P. (2003). A core collection of common bean from the iberian peninsula. *Euphytica* 131 165–175. 10.1023/A:1023973309788 30483294

[B80] Roldán M. Gómez-Mena C. Ruiz-García L. Salinas J. Martínez-Zapater J. M. (1999). Sucrose availability on the aerial part of the plant promotes morphogenesis and flowering of *Arabidopsis* in the dark. *Plant J.* 20 581–590. 10.1046/j.1365-313X.1999.00632.x 10652130

[B81] Rollins J. A. Drosse B. Mulki M. A. Grando S. Baum M. Singh M. (2013). Variation at the vernalisation genes Vrn-H1 and Vrn-H2 determines growth and yield stability in barley (*Hordeum vulgare*) grown under dryland conditions in Syria. *Theor. Appl. Genet.* 126 2803–2824. 10.1007/s00122-013-2173-y 23918065

[B82] Rosenberg N. A. (2003). distruct: a program for the graphical display of population structure. *Mol. Ecol. Notes* 4 137–138. 10.1046/j.1471-8286.2003.00566.x

[B83] Sauer N. Stolz J. (1994). SUC1 and SUC2: two sucrose transporters from *Arabidopsis thaliana*; expression and characterization in baker’s yeast and identification of the histidine-tagged protein. *Plant J.* 6 67–77. 10.1046/j.1365-313x.1994.6010067.x 7920705

[B84] Schmutz J. McClean P. E. Mamidi S. Wu G. A. Cannon S. B. Grimwood J. (2014). A reference genome for common bean and genome-wide analysis of dual domestications. *Nat. Genet.* 46 707–713. 10.1038/ng.3008 24908249 PMC7048698

[B85] Singh M. Ceccarelli S. Grando S. (1997). Precision of the genotypic correlation estimated from variety trials conducted in incomplete block designs. *TAG Theor. Appl. Genet.* 95 1044–1048. 10.1007/s001220050660

[B86] Singh M. Malhotra R. S. Ceccarelli S. Sarker A. Grando S. Erskine W. (2003). Spatial variability models to improve dryland field trials. *Exp. Agric.* 39 S0014479702001175 10.1017/S0014479702001175

[B87] Singh S. P. Gepts P. Debouck D. G. (1991a). Races of common bean (*Phaseolus vulgaris. Fabaceae*). *Econ. Bot.* 45 379–396. 10.1007/bf02887079

[B88] Singh S. P. Nodari R. Gepts P. (1991b). Genetic diversity in cultivated common bean: I. *Allozymes. Crop Sci.* 31 19–23. 10.2135/cropsci1991.0011183X003100010004x

[B89] Snape J. W. Riggs T. J. (1975). Genetical consequences of single seed descent in the breeding of self-pollinating crops. *Heredity* 35 211–219. 10.1038/hdy.1975.85

[B90] Solfanelli C. Poggi A. Loreti E. Alpi A. Perata P. (2006). Sucrose-specific induction of the anthocyanin biosynthetic pathway in *Arabidopsis*. *Plant Physiol.* 140 637–646. 10.1104/pp.105.072579 16384906 PMC1361330

[B91] TAIR (2019a). *AT1G22710.* Available at: https://www.arabidopsis.org/servlets/TairObject?type=locus&name=AT1G22710 (accessed March 25, 2019).

[B92] TAIR (2019b). *AT1G56260.* Available at: https://www.arabidopsis.org/servlets/TairObject?accession=locus:2011781 (accessed March 25, 2019).

[B93] TAIR (2019c). *AT1G65380.* Available at: https://www.arabidopsis.org/servlets/TairObject?type=locus&name=At1g65380 (accessed March 25, 2019).

[B94] TAIR (2019d). *AT4G23430.* Available at: https://www.arabidopsis.org/servlets/TairObject?type=locus&name=AT4G23430 (accessed March 25, 2019).

[B95] TAIR (2019e). *ATG22200.* Available at: https://www.arabidopsis.org/servlets/TairObject?type=locus&name=AT4G22200 (accessed March 25, 2019).

[B96] Teng S. Keurentjes J. Bentsink L. Koornneef M. Smeekens S. (2005). Sucrose-specific induction of anthocyanin biosynthesis in *Arabidopsis* requires the MYB75/PAP1 gene. *Plant Physiol.* 139 1840–1852. 10.1104/pp.105.066688.1840 16299184 PMC1310563

[B97] Tiranti B. Negri V. (2007). Selective microenvironmental effects play a role in shaping genetic diversity and structure in a *Phaseolus vulgaris* L. landrace: implications for on-farm conservation. *Mol. Ecol.* 16 4942–4955. 10.1111/j.1365-294X.2007.03566.x 17956554

[B98] United Nations. (2017). *World Population Prospect: The 2017 revision.* San Francisco: United Nations.

[B99] Visioni A. Tondelli A. Francia E. Pswarayi A. Malosetti M. Russell J. (2013). Genome-wide association mapping of frost tolerance in barley (*Hordeum vulgare* L.). *BMC Genomics* 14:424. 10.1186/1471-2164-14-424 23802597 PMC3701572

[B100] Visscher P. M. Wray N. R. Zhang Q. Sklar P. McCarthy M. I. Brown M. A. (2017). 10 Years of GWAS discovery: biology, function, and translation. *Am. J. Hum. Genet.* 101 5–22. 10.1016/j.ajhg.2017.06.005 28686856 PMC5501872

[B101] Wickham H. (2009). *Ggplot2: Elegant Graphics for Data Analysis.* Berlin: Springer.

[B102] Yan L. Hofmann N. Li S. Ferreira M. E. Song B. Jiang G. (2017). Identification of QTL with large effect on seed weight in a selective population of soybean with genome-wide association and fixation index analyses. *BMC Genomics* 18:529. 10.1186/s12864-017-3922-0 28701220 PMC5508781

[B103] Yin L. (2016). *R package “CMPlots.”.* Available at: https://github.com/YinLiLin/R-CMplot (accessed February 5, 2019).

[B104] Zander P. Amjath-Babu T. S. Preissel S. Reckling M. Bues A. Schläfke N. (2016). Grain legume decline and potential recovery in European agriculture: a review. *Agron. Sustain. Dev.* 36:26 10.1007/s13593-016-0365-y

[B105] Zeven A. C. (1997). The introduction of the common bean (*Phaseolus vulgaris* L.) into Western Europe and the phenotypic variation of dry beans collected in The Netherlands in 1946. *Euphytica* 94 319–328. 10.1023/A:1002940220241

[B106] Zhao K. Aranzana M. J. Kim S. Lister C. Shindo C. Tang C. (2007). An *Arabidopsis* example of association mapping in structured samples. *PLoS Genet.* 3:e4. 10.1371/journal.pgen.0030004 17238287 PMC1779303

