# Supplementary material for: Genome-Wide Association Study Reveals Candidate Genes for Flowering Time Variation in Common Bean (Phaseolus vulgaris L.)
Source: Front Plant Sci. 2019 Jul 24;10:962. doi: 10.3389/fpls.2019.00962 (PMC6689981; doi:10.3389/fpls.2019.00962)
Supplement: Supplementary file 1 [file Data_Sheet_1.pdf]

Genome-wide association study reveals candidate genes for flowering time variation in common bean (*Phaseolus vulgaris* L.)

Lorenzo Raggi[1a*], Leonardo Caproni[1a], Andrea Carboni[2] and Valeria Negri[1]

[1] Dipartimento di Scienze Agrarie, Alimentari e Ambientali (DSA3), Università degli Studi di Perugia, Perugia, Italy

[2] Consiglio per la Ricerca in Agricoltura e l'analisi dell'economia agraria. Centro di Ricerca Cerealicoltura e Colture Industriali (CREA-CI), Bologna, Italy

***Corresponding author:** lorenzo.raggi@unipg.it; phone number +39 075 5856212

Supplementary FIGURE 1. Boxplot of *dtf* recorded in Perugia 2016 and 2017 (PG_16 and PG_17) and Bologna 2017 (BO_17) elaborated for Andean (blue) and Mesoamerican (red) subpopulations. Assignation to the different genepools based on phaseolin information. Genotypes that did not flower were recorded as 162 days.

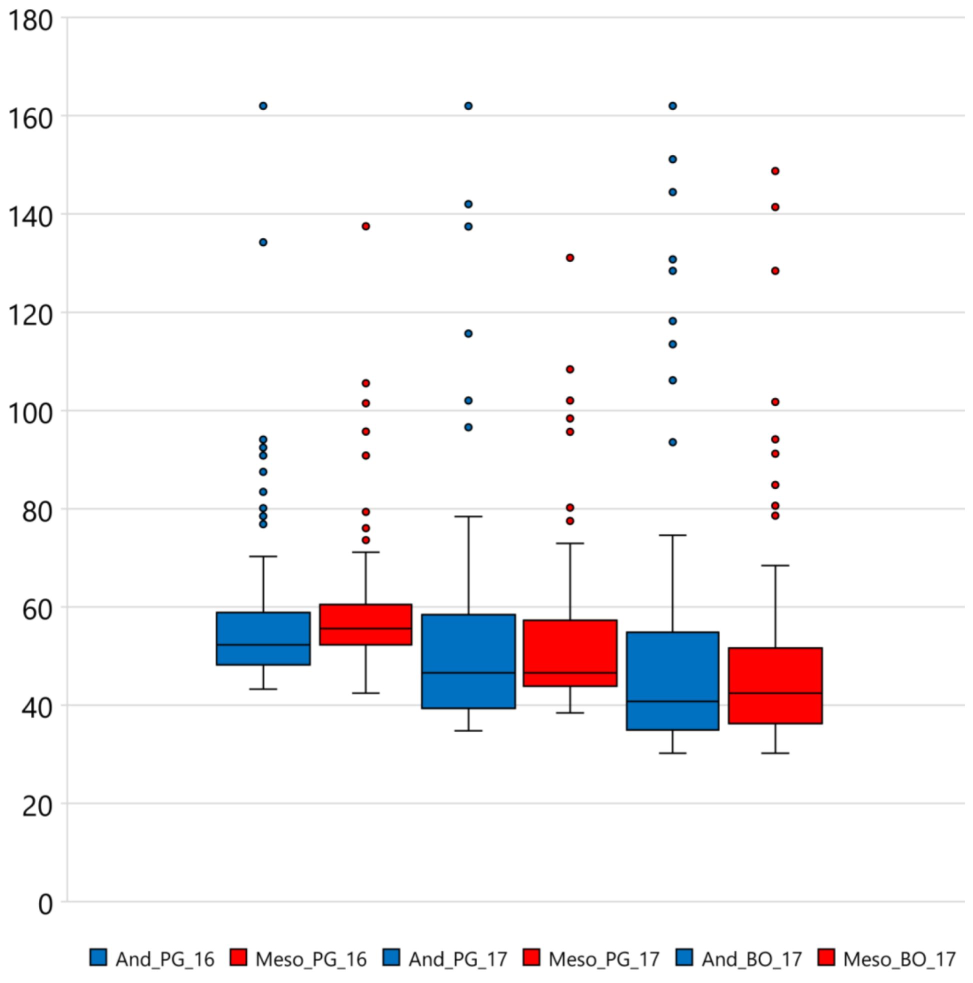

Supplementary FIGURE 2. SNP density within 1Mb window size of the reduced SNP dataset (2518) used to perform STRUCTURE and cryptic relatedness analyses; different colours represent different density levels. In the Figure "Chr" refers to common bean chromosomes.

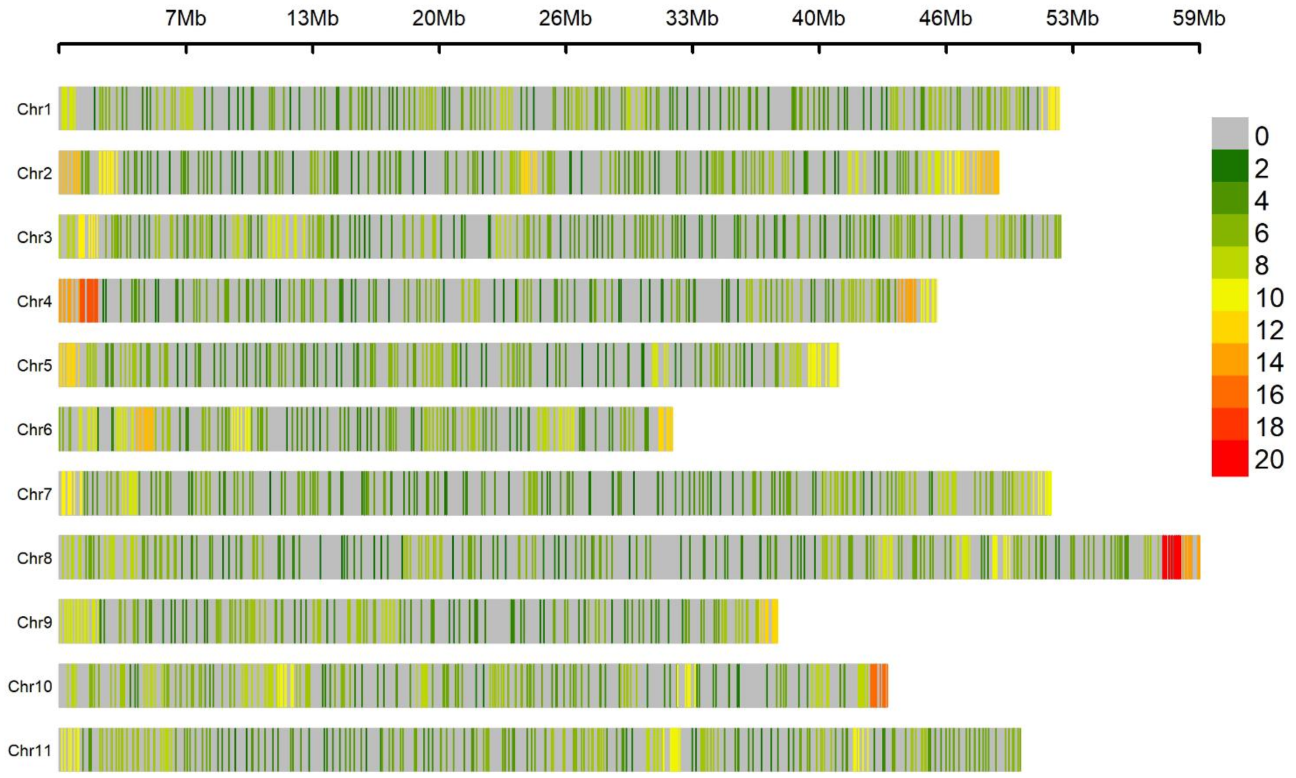

Supplementary TABLE 1. List of the 192 common bean lines developed by the Department of Agricultural, Food and Environmental Sciences (DSA3) of the University of Perugia. For each line, relevant information about the original accession, from which the line has been developed, are reported.

Line Accession Number	Original Accession Number	Collection of Reference	Common Name	Biological Status[a]	Continent[b]	Country of Origin	Growth Habit	Phaseolin Type[c]
6143	PHA2198	IPK	PHA2198	L	EU	SVK	Climbing	S
6181	PI-307786	USDA	S-215-N	L	AC	SLV	Climbing	T
6235	PI-313313	USDA	Bayo rata	L	AC	MEX	Climbing	S
6245	PI-203934	USDA	PI-203934	L	AC	MEX	Climbing	S
6246	PI-309755	USDA	Rosa de Castillo	L	AC	MEX	Climbing	S
6247	PI-312092	USDA	Frijol Garrapato	L	AC	MEX	Climbing	S
6262	W6-18758	USDA	Poroto overo	L	AS	ARG	Climbing	T
6265	W-18750	IPK	Poroto chaucha	L	AS	ARG	Prostrate- indeterm	C
6267	PI290998	USDA	Frijol Negro	L	AS	PER	Prostrate- indeterm	T
7173	G20355A	CIAT	Pesak	L	EU	MAC	Bushy- indeterm	C
7396	4646	UNIPG	O Marrozzo rampicante	L	EU	ITA	Climbing	C
7397	7185	UNIPG	Kaimano	C	EU	ITA	Climbing	NA
7398	G15440	CIAT	Cachamundinho I	L	EU	POR	Prostrate-indeterm.	S
7399	G16779	CIAT	Turkey 717-1-5	L	EU	TUR	Bushy- indeterm	T
7400	G17944	CIAT	Nyirbatori Tf 2816	L	EU	HUN	Bushy	C
7401	G20351	CIAT	Niska	L	EU	MAC	Climbing	S
7402	7181	UNIPG	Ceres	C	EU	ITA	Bushy	NA
7403	PI-151014	USDA	Amarillos	L	AS	CHI	Bushy	C
7404	PI-290995	USDA	Canario	L	AS	PER	Bushy	T
7405	G8029	CIAT	Noire D'Evian	L	EU	FRA	Bushy	C
7406	G14765	CIAT	Coco Leguyader	L	EU	FRA	Bushy	T
7407	G14786	CIAT	Le Grignorais	L	EU	FRA	Bushy	T
7408	G3613	CIAT	Alemania	L	EU	GER	Bushy	T
7409	PHA12	IPK	PHA12	L	EU	GRE	Bushy	T
7410	4652	UNIPG	Borlotto basso	L	EU	ITA	Bushy	T
7411	5071	UNIPG	Lodola precoce nano	L	EU	ITA	Bushy	T
7412	7412	UNIPG	SOLIBAM_Line_11	C	EU	ITA	Bushy	C
7413	3500	UNIPG	Cannellino Piccolo	L	EU	ITA	Bushy	C
7414	3722	UNIPG	Quarantino	L	EU	ITA	Bushy	T
7415	4299	UNIPG	San Pietro	L	EU	ITA	Bushy	T
7416	4296	UNIPG	Cannellino	L	EU	ITA	Bushy	T
7417	5079	UNIPG	Rosso lucchese	L	EU	ITA	Bushy	T
7418	G10077	CIAT	Rottekeutel	L	EU	NLD	Bushy	T
7419	G10064	CIAT	Kogelboon	L	EU	NLD	Bushy	T
7420	G10074	CIAT	Aff.Berna	L	EU	NLD	Bushy	T
7421	G10110	CIAT	Rode Kievit	L	EU	NLD	Bushy	T
7422	G15930	CIAT	Bruine Boon	L	EU	NLD	Bushy	T
7423	G18045	CIAT	Kennemer Bruine	L	EU	NLD	Bushy	T
7424	G8111	CIAT	Katarina Type 3	L	EU	POR	Bushy	T
7425	G10298	CIAT	Barrio Pole Red Montegaro	L	EU	POR	Bushy	T
7426	G387	CIAT	Kukgunuk	L	EU	TUR	Bushy	T
7427	G20427	CIAT	Oturak Fasulye	L	EU	TUR	Bushy	T
7428	MIDAS	UNIVPM	Midas	L	AS	ARG	Bushy- indeterm	T
7429	PI-156669	USDA	PI-156669	L	AS	BOL	Bushy- indeterm	C
7430	PI-352748	USDA	Gordo	L	AS	BRA	Bushy- indeterm	C
7431	PI-151015	USDA	Azulillo	L	AS	CHI	Bushy- indeterm	C
7432	PI-290989	USDA	Diacol Catio	L	AS	PER	Bushy- indeterm	T
7433	PHA3677	IPK	PHA3677	L	EU	AU	Bushy- indeterm	T
7434	PHA358	IPK	PHA358	L	EU	GRE	Bushy- indeterm	T
7435	G51731	CIAT	Long Partridge Bean	L	EU	HUN	Bushy- indeterm	T
7436	G20259	CIAT	Sut	L	EU	MAC	Bushy- indeterm	T
7438	PHA1474	IPK	PHA1474	L	EU	SVK	Bushy- indeterm	C
7439	G15790	CIAT	Banja Type, Catarino Type	L	EU	ESP	Bushy- indeterm	C
7440	G14413	CIAT	Mantecosa, Quaranta Dias	C	EU	ESP	Bushy- indeterm	T
7442	W6-17487	USDA	W6-17487	L	AS	ARG	Climbing	C
7444	W6-18792	USDA	W6-18792	L	AS	ARG	Climbing	T
7445	PI-282031	USDA	Milagros	L	AS	CHI	Climbing	T
7446	PI-282000	USDA	Pintados	C	AS	CHI	Climbing	T
7447	PHA2677	IPK	PHA2677	L	EU	BGR	Climbing	C
7448	G8013A	CIAT	Fin De Bagnols	L	EU	FRA	Climbing	T
7449	PHA3739	IPK	Saris lobio	L	EU	GEO	Climbing	C
7450	PHA303	IPK	PHA303	L	EU	GRE	Climbing	T
7452	VARI2	UNIBAS	VARI2	L	EU	ITA	Climbing	C
7453	3713	UNIPG	Borlotto bianco	L	EU	ITA	Climbing	C
7454	4362	UNIPG	Pisello bianco	L	EU	ITA	Climbing	C
7455	4959	UNIPG	Della Valle	L	EU	ITA	Climbing	C
7456	5055	UNIPG	Faxeu de Milan	L	EU	ITA	Climbing	C
7457	3506	UNIPG	Cannellino	L	EU	ITA	Climbing	T
7458	4102	UNIPG	Bianco	L	EU	ITA	Climbing	T
7459	4294	UNIPG	Mezza Rama	L	EU	ITA	Climbing	C
7460	4361	UNIPG	Borlotto chiaro da minestra	L	EU	ITA	Climbing	C
7461	4455	UNIPG	A Pane	L	EU	ITA	Climbing	C
7462	4458	UNIPG	Aquilano	L	EU	ITA	Climbing	C
7463	4554	UNIPG	Carnaccia	L	EU	ITA	Climbing	C
7464	5040	UNIPG	Borlotto	L	EU	ITA	Climbing	C
7465	5047	UNIPG	Laurons	L	EU	ITA	Climbing	C
7466	5049	UNIPG	Militons	L	EU	ITA	Climbing	T

7467	5053	UNIPG	Ballin de loto	L	EU	ITA	Climbing	C
7468	5084	UNIPG	Del gaggio	L	EU	ITA	Climbing	T
7469	4087	UNIPG	Borlotto nano	L	EU	ITA	Climbing	C
7470	4956	UNIPG	Meraviglia	L	EU	ITA	Climbing	C
7471	G20110A	CIAT	Cucavec	L	EU	MAC	Climbing	T
7472	G10100	CIAT	Stokkievitsboon	L	EU	NLD	Climbing	T
7473	G15921	CIAT	Snijboon	L	EU	NLD	Climbing	T
7474	G10234	CIAT	Pole Yellowish Buff	L	EU	POR	Climbing	T
7475	G10230	CIAT	Pole Deep Purple Kidney	L	EU	POR	Climbing	C
7476	PHA3065	IPK	PHA3065	L	EU	ROU	Climbing	C
7477	G13936	CIAT	Don Timoteo	L	EU	ESP	Climbing	C
7478	G1029	CIAT	Herraduca Argentinos	L	EU	ESP	Climbing	C
7479	G10154	CIAT	Dwarf Redonda	L	EU	ESP	Climbing	C
7480	G461	CIAT	Maras	L	EU	TUR	Climbing	T
7481	G316	CIAT	Barbunya	L	EU	TUR	Climbing	C
7482	Jalo EEP558	UNIBAS	Jalo EEP558	L	AS	ARG	Prostrate- indeterm	T
7483	G13765	CIAT	Anadoran	L	AS	BRA	Prostrate- indeterm	C
7485	PHA2134	IPK	PHA2134	L	EU	GEO	Prostrate- indeterm	T
7486	G11826	CIAT	Mohacsi Lila Furj	L	EU	HUN	Prostrate- indeterm	T
7487	PHA2360	IPK	PHA2360	L	EU	ITA	Prostrate- indeterm	T
7488	G10062	CIAT	Aff.Berna (V.Namen)	L	EU	NLD	Prostrate- indeterm	T
7489	G15443	CIAT	Pole Large Seeded R.Kidney	L	EU	POR	Prostrate- indeterm	T
7490	PHA3063	IPK	PHA3063	L	EU	ROU	Prostrate- indeterm	T
7491	G14405	CIAT	Burros Argentinos Type	L	EU	ESP	Prostrate- indeterm	C
7492	NGB9299.3	NGB	NGB9299.3	C	EU	SWE	Prostrate- indeterm	T
7493	G15856	CIAT	Yenidunya Fasulya	L	EU	TUR	Prostrate- indeterm	C
7494	4293	UNIPG	Zolferino	L	EU	ITA	NA	T
7495	4916	UNIPG	Borlotto nano	L	EU	ITA	Bushy	T
7496	4110	UNIPG	Zolferino	L	EU	ITA	NA	T
7497	G17830A	CIAT	Fulokercsi Tf 2608	L	EU	HUN	Bushy	S
7498	4616	UNIPG	Di Onano Gradoli	L	EU	ITA	Bushy	S
7499	4406	UNIPG	Del Purgatorio	L	EU	ITA	Bushy	S
7500	4135	UNIPG	Nero	L	EU	ITA	Bushy	S
7501	G11151	CIAT	Perle	L	EU	Lithuania	Bushy	S
7502	G3763	CIAT	Noire Hatif De Belgique	L	EU	Belgium	Bushy-indeterm.	S
7503	PHA1781	IPK	PHA1781	L	EU	GER	Bushy-indeterm.	S
7504	5069	UNIPG	Bianco con tralcio senza frasca	L	EU	ITA	Bushy-indeterm.	S
7505	G10112	CIAT	Citroen (Lpt)	L	EU	NLD	Bushy-indeterm.	S
7506	G10210	CIAT	Dwarf Port Viana	L	EU	POR	Bushy-indeterm.	S
7507	G10252	CIAT	Cachamundinho Semi-Climb.	L	EU	POR	Bushy-indeterm.	S
7508	G993	CIAT	Alubia Pinta De Leon	L	EU	ESP	Bushy-indeterm.	S
7509	G11573	CIAT	Avellaneta Negra, 375 Vol Niger	L	EU	ESP	Bushy-indeterm.	S
7510	NGB11752.2	NGB	NGB11752.2	L	EU	SWE	Bushy-indeterm.	S
7511	G900	CIAT	Ohlsenia Oe/47	L	EU	SWE	Bushy-indeterm.	S
7512	G2925	CIAT	Selection From Pi204715	L	EU	TUR	Bushy-indeterm.	S
7513	G16741	CIAT	Turkey Bursa 12c	L	EU	TUR	Bushy-indeterm.	S
7514	G1018	CIAT	Black Mexican	L	AC	MEX	Bushy-indeterm.	S
7515	PI-309837	USDA	Frijo chileno y colorado	L	AC	CRI	Climbing	S
7516	PI-309831	USDA	Frijol Carne	L	AC	CRI	Climbing	S
7517	PI-309885	USDA	Chimbolos	L	AC	CRI	Climbing	S
7518	PI-311794	USDA	Tineco Rojo	L	AC	SLV	Climbing	S
7519	PI-304113	USDA	Ch-60-111-2	L	AC	SLV	Climbing	S
7520	PI-310586	USDA	Sangre de Toro	L	AC	HND	Climbing	S
7525	PI-416407	USDA	Jamapa	C	AC	MEX	Climbing	S
7526	PI-583642	USDA	Carioca	L	AS	BRA	Climbing	S
7527	PI-151017	USDA	Bayos Palos	L	AS	CHI	Climbing	S
7528	PI-152326	USDA	Negro Redondo	L	AS	ECU	Climbing	S
7529	PI-306149	USDA	Caraota	L	AS	PER	Climbing	S
7530	PHA6857	IPK	PHA6857	L	EU	ALB	Climbing	S
7531	PHA5913	IPK	Barbunjë	L	EU	ALB	Climbing	S
7532	PHA6266	IPK	PHA6266	L	EU	ALB	Climbing	S
7533	PHA3640	IPK	PHA3640	L	EU	AU	Climbing	S
7534	PHA7079	IPK	Barbunjë	L	EU	BGR	Climbing	S
7535	09L0506251	UNIBAS	NA	L	EU	CZE	Climbing	S
7536	PHA2786	IPK	PHA2786	L	EU	GEO	Climbing	S
7537	PHA7002	IPK	PHA7002	L	EU	GER	Climbing	S
7538	58431SemeBia	UNIBAS	58431 Seme Bianco	L	EU	GER	Climbing	S
7539	PHA285	IPK	PHA285	L	EU	GRE	Climbing	S
7540	PHA3927	IPK	PHA3927	L	EU	HUN	Climbing	S
7541	PHA1933	IPK	PHA1933	L	EU	ITA	Climbing	S
7542	PHA1916	IPK	PHA1916	L	EU	ITA	Climbing	S
7543	5058	UNIPG	Lambardèn	L	EU	ITA	Climbing	S
7544	4295	UNIPG	Grigio	L	EU	ITA	Climbing	S
7545	4459	UNIPG	Mangiatutto	L	EU	ITA	Climbing	S
7546	5080	UNIPG	Staiccioli	L	EU	ITA	Climbing	S
7547	3726	UNIPG	Cannellino	L	EU	ITA	Climbing	S
7548	4651	UNIPG	Fagiolo Alto al Burro	L	EU	ITA	Climbing	S
7549	5057	UNIPG	Cesarins	L	EU	ITA	Climbing	S
7550	G20087	CIAT	Edar Visek	L	EU	MAC	Climbing	S
7551	G20109	CIAT	Krupen	L	EU	MAC	Climbing	S
7552	G20291	CIAT	Nizok	L	EU	MAC	Climbing	S
7553	PHA1684	IPK	PHA1684	L	EU	POL	Climbing	S
7554	G10241	CIAT	Pole Par.De Cowa White Lrg	L	EU	POR	Climbing	S
7555	G10248A	CIAT	Semiclimber Var. Flat Pods	L	EU	POR	Climbing	S
7557	G14415	CIAT	G14415	L	EU	ESP	Climbing	S
7558	G15293	CIAT	White Coco Type	L	EU	ESP	Climbing	S
7559	PHA130	IPK	PHA130	L	EU	TUR	Climbing	S
7560	G15618	CIAT	Oturak Fasulya	L	EU	TUR	Climbing	S

7561	G309	CIAT	Aysekadin	L	EU	TUR	Climbing	S
7562	PHA5937	IPK	PHA5937	L	EU	UKR	Climbing	S
7563	PI-309842	USDA	Chimbolo	L	AC	CRI	Prostrate-indeterm.	S
7564	PI-308891	USDA	Frijol amarillo	L	AC	CRI	Prostrate-indeterm.	S
7565	PI-345574	USDA	Jamapa	L	AC	CRI	Prostrate-indeterm.	S
7566	PI-196927	USDA	Frijol Criollo	L	AC	MEX	Prostrate-indeterm.	S
7567	PI-300668	USDA	Negro Argel	L	AS	CHI	Prostrate-indeterm.	S
7568	PI-313888	USDA	Col. No. 65	C	AS	VEN	Prostrate-indeterm.	S
7569	G20198A	CIAT	Bel Nizok	L	EU	HRV	Prostrate-indeterm.	S
7570	G20238	CIAT	Lokvicka	L	EU	HRV	Prostrate-indeterm.	S
7571	G15927	CIAT	Fijne Kleine Boon	L	EU	NLD	Prostrate-indeterm.	S
7572	G11854	CIAT	Walcherse Witte (Lpt)	L	EU	NLD	Prostrate-indeterm.	S
7573	G14699	CIAT	Seleccion De Mezcla Ix	L	EU	POR	Prostrate-indeterm.	S
7574	G10164	CIAT	Long White Kidney Tipe-Pol	L	EU	ESP	Prostrate-indeterm.	S
7575	G15231	CIAT	Riñon Pequeña	L	EU	ESP	Prostrate-indeterm.	S
7576	G15574	CIAT	Beyaz Fasulya	L	EU	TUR	Prostrate-indeterm.	S
7577	G15881	CIAT	Yer Fasulyasi	L	EU	TUR	Prostrate-indeterm.	S
7578	4914	UNIPG	Bianco	L	EU	ITA	NA	S
7579	6388	UNIPG	St. Esprit	L	EU	FRA	Bushy	NA
7580	6389	UNIPG	Flageolet Chevrier	L	EU	FRA	Bushy	NA
7581	6390	UNIPG	Rognon de Coq	L	EU	FRA	Bushy	NA
7582	7582	UNIPG	Magico (Borlotto)	C	EU	ITA	Bushy	NA
7583	7583	UNIPG	Telemaco (Mangiatutto)	C	EU	ITA	Bushy	NA
7584	6387	UNIPG	Roi de Belges	L	EU	FRA	Bushy	NA
7585	7585	UNIPG	White Top (Cannellino)	C	EU	ITA	Bushy	NA
7586	7586	UNIPG	Dipinto (Borlotto)	C	EU	ITA	Climbing	NA
7587	7587	UNIPG	Kondor (Cannellino)	C	EU	ITA	Climbing	NA

[a]C=cultivar; L=landrace. [b]AC=America, Central; AS=America, South; EU=Europe. [c]NA=Not Available.

Supplementary TABLE 2. BLUPs dataset of days to flowering.

Line (Accession Number)	PG-2016	PG-2017	BO-2017
6143	50.64	46.59	44.18
6181	162.00	162.00	162.00
6235	105.55	108.38	141.43
6245	90.80	80.21	101.73
6246	137.52	131.09	148.75
6247	96.54	102.02	128.42
6262	NA	142.00	130.77
6265	NA	39.32	36.85
6267	NA	102.93	144.46
7173	NA	115.65	128.42
7396	84.44	69.46	73.44
7397	63.18	74.20	84.77
7398	57.62	56.32	80.60
7399	70.26	52.60	54.17
7400	65.47	48.48	40.77
7401	57.68	57.25	40.76
7402	46.11	46.93	33.57
7403	NA	38.42	32.56
7404	87.52	NA	NA
7405	51.46	44.78	36.85
7406	NA	46.59	36.02
7407	46.54	35.69	32.56
7408	47.36	36.60	34.92
7409	53.92	52.95	37.95
7410	44.90	35.69	32.56
7411	44.90	38.42	34.92
7412	45.72	38.42	34.92
7413	58.02	40.23	36.02
7414	43.26	34.78	33.95
7415	49.82	40.23	38.92
7416	48.18	40.23	37.82
7417	48.18	38.42	34.50
7418	45.72	35.69	30.21
7419	44.90	36.60	35.47
7420	50.64	36.60	30.21
7421	47.36	40.23	34.92
7422	55.56	38.42	33.53
7423	46.54	38.42	31.18
7424	44.90	40.23	32.56
7425	46.54	37.51	36.85
7426	92.44	96.57	106.15
7427	49.00	59.31	42.80
7428	53.10	41.14	36.02
7429	49.00	38.42	31.18
7430	80.14	62.95	57.32
7431	77.69	45.68	54.83
7432	47.36	44.78	55.80

7433	54.74	41.14	33.95
7434	44.90	36.60	31.59
7435	52.28	55.68	46.12
7436	44.90	38.42	33.95
7438	46.54	42.05	36.02
7439	55.56	48.41	57.32
7440	50.64	36.60	34.50
7442	134.24	137.45	151.11
7444	135.06	138.36	145.30
7445	90.80	72.03	118.19
7446	50.64	38.42	36.02
7447	53.10	54.77	37.95
7448	NA	NA	NA
7449	77.69	62.04	47.50
7450	54.74	46.59	46.12
7452	52.28	55.68	40.73
7453	50.64	62.95	50.96
7454	49.82	46.59	40.73
7455	59.65	70.22	72.26
7456	50.64	52.95	40.73
7457	53.10	42.96	41.83
7458	54.74	46.59	51.51
7459	49.00	59.31	39.89
7460	45.72	54.77	40.73
7461	49.00	61.13	43.63
7462	54.74	62.04	57.32
7463	NA	102.02	113.48
7464	53.10	54.77	74.62
7465	58.83	58.41	56.22
7466	50.64	47.50	38.37
7467	67.03	60.22	46.67
7468	53.10	46.59	42.80
7469	53.92	39.32	36.85
7470	49.82	39.32	38.37
7471	53.92	42.96	42.24
7472	58.83	55.68	44.18
7473	55.56	47.50	40.73
7474	51.46	42.96	38.37
7475	55.56	52.95	43.63
7476	53.10	51.14	44.73
7477	94.08	78.39	113.48
7478	76.87	46.59	45.15
7479	NA	45.68	38.37
7480	80.96	72.03	93.56
7481	51.46	53.86	41.83
7482	83.42	58.41	69.91
7483	54.74	52.05	55.80
7485	52.28	42.05	36.02
7486	55.56	52.95	66.03

7487	46.54	42.96	34.92
7488	49.00	38.42	33.53
7489	NA	56.59	69.91
7490	62.93	43.87	43.63
7491	52.28	47.50	40.73
7492	45.72	37.51	32.56
7493	78.51	58.41	38.92
7494	50.64	43.87	38.92
7495	44.90	38.42	31.18
7496	48.18	42.96	33.53
7497	49.00	41.14	34.50
7498	61.29	42.96	35.47
7499	52.28	44.78	36.02
7500	47.36	47.50	43.63
7501	53.10	47.50	36.02
7502	49.82	44.78	39.89
7503	95.72	98.38	94.11
7504	52.28	46.59	38.37
7505	52.28	42.96	40.73
7506	76.05	44.78	44.18
7507	52.28	NA	91.21
7508	NA	47.50	62.03
7509	57.20	47.50	43.63
7510	47.36	41.14	34.92
7511	53.10	41.14	36.85
7512	55.56	61.13	30.21
7513	61.29	55.68	54.83
7514	63.75	65.67	52.35
7515	61.29	58.41	46.54
7516	42.44	48.41	49.44
7517	79.33	59.31	68.39
7518	57.20	45.68	42.24
7519	NA	65.67	58.16
7520	71.13	46.59	41.83
7525	57.20	48.41	40.73
7526	54.74	45.68	37.95
7527	51.46	42.05	40.73
7528	57.20	46.59	42.66
7529	73.59	66.58	59.67
7530	52.28	42.05	39.89
7531	51.46	41.14	36.02
7532	54.74	43.87	44.73
7533	52.28	45.68	42.66
7534	52.28	40.23	35.47
7535	53.10	46.59	36.02
7536	101.45	95.66	84.85
7537	58.83	39.32	36.02
7538	52.28	40.23	34.50
7539	52.28	40.23	33.11

7540	52.28	45.68	42.24
7541	60.47	46.59	45.15
7542	63.75	45.68	39.89
7543	55.56	54.77	45.15
7544	52.28	44.78	34.50
7545	55.56	48.41	41.28
7546	55.56	65.67	58.16
7547	53.10	48.41	42.66
7548	46.54	38.42	31.18
7549	49.82	39.32	31.18
7550	65.39	48.41	48.60
7551	55.56	59.31	78.62
7552	52.28	40.23	34.50
7553	58.02	52.05	46.12
7554	66.21	69.31	58.16
7555	62.11	77.49	52.48
7557	53.10	39.32	33.53
7558	49.82	44.78	43.63
7559	60.47	59.31	68.39
7560	55.56	46.59	36.02
7561	55.56	72.03	59.67
7562	52.28	44.78	37.95
7563	76.05	72.94	61.61
7564	58.02	44.78	39.89
7565	58.83	46.59	42.66
7566	55.56	46.59	42.24
7567	54.74	55.68	44.60
7568	57.20	46.59	38.92
7569	49.82	47.50	36.85
7570	49.82	39.32	35.47
7571	56.38	59.31	47.50
7572	52.28	39.32	38.37
7573	55.56	47.50	37.95
7574	54.74	43.87	42.80
7575	52.28	51.14	34.92
7576	44.90	39.32	33.95
7577	NA	43.87	46.67
7578	55.56	45.68	46.12
7579	40.80	40.23	36.85
7580	47.36	41.14	37.95
7581	52.28	40.23	32.56
7582	44.90	36.60	33.11
7583	44.90	35.69	32.56
7584	44.90	39.32	32.56
7585	45.72	37.51	32.56
7586	49.82	53.86	43.63
7587	54.74	48.41	42.80
